# Structural insights into histone exchange by human SRCAP complex

**DOI:** 10.1038/s41421-023-00640-1

**Published:** 2024-02-08

**Authors:** Jiali Yu, Fengrui Sui, Feng Gu, Wanjun Li, Zishuo Yu, Qianmin Wang, Shuang He, Li Wang, Yanhui Xu

**Affiliations:** 1 Fudan University Shanghai Cancer Center, Institutes of Biomedical Sciences, New Cornerstone Science Laboratory, State Key Laboratory of Genetic Engineering and Shanghai Key Laboratory of Medical Epigenetics, Shanghai Medical College of Fudan University, Shanghai, China; 2 The International Co-laboratory of Medical Epigenetics and Metabolism, Ministry of Science and Technology of China, Department of Systems Biology for Medicine, School of Basic Medical Sciences, Shanghai Medical College of Fudan University, Shanghai, China; 3 Greater Bay Area Institute of Precision Medicine, Fudan University, Nansha District, Guangzhou, Guangdong China

**Keywords:** Chromatin remodelling, Cryoelectron microscopy

## Abstract

Histone variant H2A.Z is found at promoters and regulates transcription. The ATP-dependent chromatin remodeler SRCAP complex (SRCAP-C) promotes the replacement of canonical histone H2A–H2B dimer with H2A.Z–H2B dimer. Here, we determined structures of human SRCAP-C bound to H2A-containing nucleosome at near-atomic resolution. The SRCAP subunit integrates a 6-subunit actin-related protein (ARP) module and an ATPase-containing motor module. The ATPase-associated ARP module encircles half of the nucleosome along the DNA and may restrain net DNA translocation, a unique feature of SRCAP-C. The motor module adopts distinct nucleosome binding modes in the apo (nucleotide-free), ADP-bound, and ADP-BeF_x_-bound states, suggesting that ATPase-driven movement destabilizes H2A–H2B by unwrapping the entry DNA and pulls H2A–H2B out of nucleosome through the ZNHIT1 subunit. Structure-guided chromatin immunoprecipitation sequencing analysis confirmed the requirement of H2A-contacting ZNHIT1 in maintaining H2A.Z occupancy on the genome. Our study provides structural insights into the mechanism of H2A-H2A.Z exchange mediated by SRCAP-C.

## Introduction

In eukaryotic cells, genomic DNA wraps around histone octamers to generate arrayed nucleosomes on the genome. A canonical nucleosome contains two copies of the four histones H2A, H2B, H3, and H4^1^. To achieve functional regulation, ATP-dependent chromatin remodeling complexes (remodelers) change nucleosome composition and position along the DNA^2,3^. Chromatin remodeling complexes can be divided into four families according to the similarities and differences in their catalytic ATPases: imitation switch (ISWI), chromodomain helicase DNA-binding (CHD), switch/sucrose non-fermentable (SWI/SNF) and Inositol auxotrophy 80 (INO80). Two of these remodelers comprise either a single subunit (CHD family) or a few subunits (ISWI family). By contrast, the SWI/SNF and INO80 families are multi-subunit complexes that, in addition to a superfamily II helicase-like motor subunit, contain actin and actin-related proteins (ARPs) and other remodeler-specific subunits with different functions^2,4,5^.

The human chromatin remodeler SNF2-related CPB activator protein (SRCAP) complex (SRCAP-C)^6,7^ and its yeast counterpart SWR1 complex (SWR-C)^8^ belong to the INO80 chromatin remodeler family^4,9^. SRCAP-C and SWR-C could replace canonical H2A–H2B dimer with H2A.Z–H2B dimer in an ATP-dependent manner^10–15^ and this process may require histone chaperones^16–18^. The generated H2A.Z-containing nucleosomes are enriched at promoter regions of nearly all genes in euchromatin^13,19–21^ and are involved in the regulation of transcription and other processes such as DNA replication, DNA repair, and chromosome segregation^22–30^. In eukaryotic cells, H2A.Z is predominantly found at the distal end of the promoter and plays a critical role in RNAPII initiation and elongation^23,24,30^. H2A.Z is also broadly enriched at replication origins and has been directly linked to DNA replication by regulating the initiation of early replication origins and replication timing^26^. Moreover, studies have revealed that coordinated H2A.Z dynamics at double-strand breaks (DSBs) is essential for DNA repair^27,28^. H2A.Z also plays a crucial role in the chromosome segregation process to maintain the genome in a stable state^29^. Distinct from other chromatin remodelers^2,4^, SWR-C binds to nucleosome and induces DNA unwrapping and rewrapping^14,31,32^, but does not generate net DNA translocation^14,33^, exhibiting a characteristic feature.

**Fig. 1:**
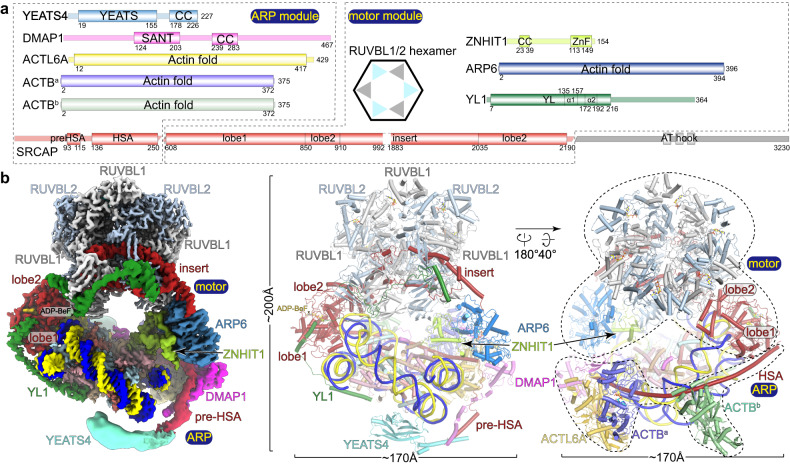
Overall structure of the nucleosome-bound SRCAP-C. **a** Domain structure of the human SRCAP-C. The ARP and motor modules are indicated with dashed boxes. There are two copies of ACTB in the SRCAP-C, called ACTB^a^ and ACTB^b^. Color scheme is used throughout figures if not elsewhere specified. **b** Composite cryo-EM map and structural model of the nucleosome-bound SRCAP-C in the ADP-BeF_x_-bound state. Right model shows that the motor and ARP modules merge at the ATPase domain of the SRCAP subunit.

The human SRCAP-C is an ~1-MDa complex consisting of 10 subunits including SRCAP, YL1, RUVBL1, RUVBL2, ARP6, ZNHIT1, DMAP1, ACTL6A, ACTB, and YEATS4 (Fig. 1a), and the yeast SWR-C consists of 10 equivalent subunits, Swr1, Swc2, RvB1, RvB2, Arp6, Swc6, Swc4, Arp4, Actin, Yaf9 and 4 additional yeast-specific subunits, Swc3, Swc5, Swc7 and Bdf1^8,34,35^. Previous studies have revealed structures of yeast SWR-C^15,36^, human SRCAP-C^37^, and nucleosome-bound yeast SWR-C in the presence of ADP-BeF_x_, an ATP analog^14^. SWR-C contains a heterohexamer of the RuvBL proteins, with the insert in Swr1 extending through the RuvBL hexamer. The ATPase domain of Swr1 grasps DNA at superhelical location (SHL) 2, causing translocation of the DNA by 1 bp in the ADP-BeF_x_-bound state. In addition, entry DNA is partially unwrapped, and the histone core “flexes”. These conformational changes suggest a mechanism of H2A.Z exchange without net translocation of DNA in yeast^14^. SRCAP-C adopts a generally similar architecture compared to yeast SWR-C, containing a ring-shaped “head” with two separate arms^37^.

Despite these studies, due to the lack of structures of ADP-bound SWR-C–nucleosome and SRCAP-C–nucleosome, the mechanism by which SRCAP-C promotes H2A-H2A.Z exchange remains incompletely understood. Here, we determined the structures of nucleosome-bound SRCAP-C in different nucleotide-bound states and performed structure-guided chromatin immunoprecipitation sequencing (ChIP-seq) analysis to test the effect of ZNHIT1 on H2A.Z incorporation in vivo, shedding light on the mechanism of H2A-H2A.Z exchange mediated by SRCAP-C.

## Results

### Complex assembly and structure determination

To understand the mechanism of histone exchange by SRCAP-C, we first overexpressed the 10-subunit human SRCAP complex in 293F suspension cells and purified the complex to homogeneity (Fig. 1a; Supplementary Fig. S1a). However, no histone exchange activity was detected using the highly purified 10-subunit complex in our experimental condition (data not shown), possibly due to the absence of necessary factors yet to be discovered in human cells. For example, Swc5 in SWR-C is required for histone exchange activity^15,38^, whereas no equivalent subunit exists in the 10-subunit SRCAP-C. In an earlier study^11^, the SRCAP-containing complex possessing H2A-H2A.Z exchange activity was purified from HeLa cells, which may contain factors necessary for exchange.

The absence of histone exchange activity could also result from the lack of ATPase activity in the SRCAP subunit. We next performed the ATPase activity assay using the purified SRCAP-C with the Polybromo, Brg1/Brm-associated factor (PBAF) complex as a control. The purified SRCAP-C showed weak but noticeable ATPase activity in the absence of substrate (Supplementary Fig. S1b). Upon the addition of nucleosome substrate, the ATPase activity of PBAF complex increased by ~8-fold^39^, whereas the activity of SRCAP-C increased by ~2-fold, comparable to the previous observation of SWR-C^40^. We did not observe evident stimulation of ATPase activity by H2A.Z–H2B dimer^15,40^. To test whether the ATPase activity of SRCAP-C is derived from SRCAP ATPase or RUVBL1–RUVBL2 AAA+ ATPases hexamer^41^, we purified an SRCAP-C containing an SRCAP ATPase-dead mutant with key residues in the ATP-binding pocket mutated (K649G, R2151G, R2154G). The mutant SRCAP-C showed largely decreased ATPase activity compared to the wild-type (WT) SRCAP-C, and this activity could not be further stimulated by nucleosome (Supplementary Fig. S1b). This result indicates that SRCAP subunit has ATPase activity and could be stimulated by nucleosome, whereas the RUVBL1–RUVBL2 hexamer may contribute to the remaining low ATPase activity.

Studies have revealed that the H2A-containing nucleosome is a substrate for human SRCAP-C and yeast SWR-C^10–13^, and that nucleosome flanked by a long DNA fragment is a more favorable substrate^31,42^. SWR-C and SRCAP-C are mainly enriched at +1 nucleosome^10,12,42^, which is flanked by a long nucleosome-free region and a short gene body linker^20,43^. Thus, we assembled SRCAP-C with a nucleosome core particle (NCP) containing long (108 bp) and short (12 bp) flanking DNA fragments (termed 108N12) mimicking the in vivo substrate of SRCAP-C. The complex is assembled in the presence of ADP and ADP-BeF_x_, respectively, and subjected to gradient fixation and structure determination by cryo-electron microscopy (cryo-EM) single-particle reconstruction (Fig. 1b; Supplementary Fig. S1c, d and Table S1).

ADP-BeF_x_ is a structural analog of the pre-ATP hydrolysis nucleotide, where BeF_x_ mimics γ-phosphoryl group in the ground state. ADP and beryllium fluoride together tend to bind to ATP-binding sites and inhibit ATPase action, locking the complex in an activated state^44^. Cryo-EM 3D classification showed conformations in the apo (nucleosome-bound and nucleotide-free) and ADP-bound states in the ADP-containing sample and conformations in the apo and ADP-BeF_x_-bound states in the ADP-BeF_x_-containing sample, respectively (Supplementary Fig. S1c). The cryo-EM map in the ADP-BeF_x_-bound conformation shows that ADP, but not ADP-BeF_x_, is present in RUVBL1 and RUVBL2, consistent with the remaining low ATPase activity of the ATPase-dead mutant of SRCAP-C (Supplementary Fig. S1b). This observation is similar to that in SWR-C structure^14^. The cryo-EM maps were refined to an overall resolution of ~3.3 Å and maps of the majority of subcomplexes were locally refined to resolution of 2.9–4.2 Å. Structural models of the three complexes were built according to the cryo-EM maps aided by crosslinking mass spectrometry (XLMS) analysis (Supplementary Fig. S2 and Table S2).

Despite the use of a high concentration of either ADP or ADP-BeF_x_ nucleotide in the sample along with a crosslinker, the majority of the selected particles in both cases correspond to the apo state of the complex, possibly due to the following reasons. (1) Glutaraldehyde generates crosslinks between lysine residues but not nucleotides. The ADP-bound state of SRCAP-C–NCP is a post-hydrolysis state, where the ADP tends to dissociate from the ATP-binding site. (2) ADP-BeF_x_, a structural analog of ATP, may not efficiently bind the ATP-binding site. For example, single-molecule FRET analysis of nucleosome–SWR-C interaction showed much higher dynamic traces in the presence of ATP than in the presence of ADP-BeF_x_^14^. The generation of particles of ADP-BeF_x_-bound INO80 C-module with nucleosome is also less efficient^45^. (3) SRCAP-C shows low ATPase activity compared to other remodelers^40^ (Supplementary Fig. S1b). Thus, particles in the apo state may reflect dissociation and/or unsuccessful binding of nucleotides during sample preparation.

### Overall structure of the nucleosome-bound SRCAP-C

Cryo-EM map of the complex in the ADP-BeF_x_-bound state reveals a compact fold with approximate dimensions of ~170 Å × 170 Å × 200 Å (Fig. 1; Supplementary Video S1). Nucleosome and SRCAP-C are organized in 1:1 stoichiometry. SRCAP-C can be divided into motor and ARP modules, which merge at the ATPase of the SRCAP subunit. As a scaffold, the SRCAP subunit consists of an N-terminal helicase-SANT-associated (HSA) and pre-HSA helices that integrate the ARP module, an ATPase domain grasping the nucleosomal DNA, and an insert threading through the RUVBL1–RUVBL2 hexamer. The motor module consists of RUVBL1–RUVBL2 hexamer, YL1, ARP6, ZNHIT1, and the majority of the SRCAP subunit. The ARP module consists of DMAP1, ACTL6A, two ACTB subunits, YEATS4, and the HSA and pre-HSA helices of SRCAP subunit. The motor module adopts a similar fold to those of human SRCAP-C in the absence of substrate^37^ and nucleosome-bound yeast SWR-C^14^, whereas the 6-subunit ARP module has not been previously observed in the context of SRCAP-C or SWR-C (Supplementary Fig. S3a, b).

### The motor module and its binding to nucleosome

**Fig. 2:**
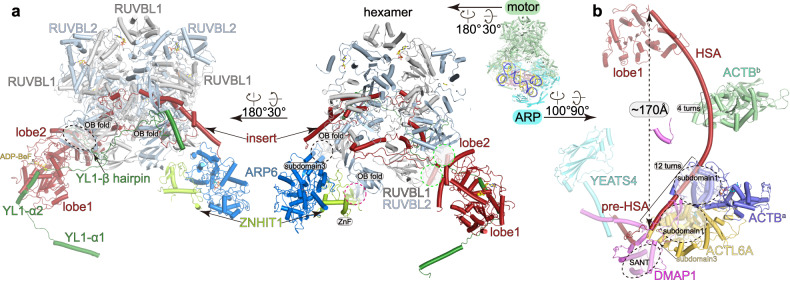
Structures of the motor and ARP modules. **a**, **b** Structural models of the motor (**a**) and ARP (**b**) modules showing the interactions of subunits, with interactions indicated by dashed circles.

**Fig. 3:**
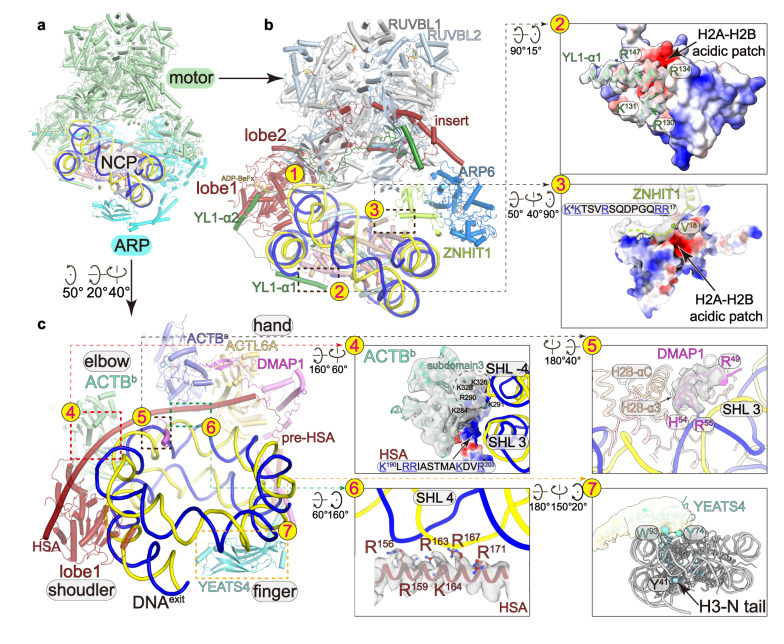
Interactions between nucleosome and SRCAP-C. **a** Overall structure of the nucleosome-bound SRCAP-C with the motor and ARP modules colored in green and cyan, respectively. **b**, **c** The motor (**b**) and ARP (**c**) modules make multiple contacts with nucleosome. Left panels show the positions of the interfaces and right panels show close-up views. Cryo-EM maps of nucleosome-binding regions of SRCAP subunits are shown in transparent surfaces and structural models. The motor-proximal and motor-distal H2A–H2B dimers are shown in electrostatic surfaces.

Within the motor module, the SRCAP insert is embedded within the RUVBL1–RUVBL2 hexamer, creating a central core that associates with two nucleosome-binding branches, the SRCAP ATPase domain and the ARP6–ZNHIT1 heterodimer (Fig. 2a; Supplementary Fig. S4b). The ATPase grasps the nucleosomal DNA at SHL 2 with the lobe1 interacting with the long HSA helix of the ARP module (interface-1) (Fig. 3b). The ATPase lobe2 directly merges with the RUVBL1–RUVBL2 hexamer at two edges of the SRCAP insert, generating a stable association between the ATPase and RUVBL1–RUVBL2 hexamer. ARP6 and ZNHIT1 form a heterodimer and make extensive contact with the RUVBL1–RUVBL2 hexamer. ZNHIT1 binds an OB-fold of an RUVBL2 through its zinc finger (ZnF) domain. The subdomain3 of ARP6 contacts an OB-fold of a nearby RUVBL1 and an α-helix of the SRCAP insert (Fig. 2a).

YL1 adopts an extended conformation and winds over the surfaces of the nucleosome, the ATPase, and the RUVBL1–RUVBL2 hexamer (Figs. 2a, 3b). The N-terminal helix α1 packs on the surface of the motor-distal H2A–H2B dimer (interface-2) (Fig. 3b). The following helix α2 binds the ATPase lobe1. A β hairpin fills in a gap between two OB-folds of an RUVBL1 and an RUVBL2. The C-terminal mixed α/β motif binds a nearby OB-fold of an RUVBL2 and contacts the exposed region of the SRCAP insert.

The motor module embraces one entire gyre of the nucleosome (Fig. 3b; Supplementary Fig. S4b). While the overall fold is similar to the structure of nucleosome-bound yeast SWR-C^14^ (Supplementary Fig. S3a, b), we observed the following contacts that were not previously defined. At interface-3 (Fig. 3b), we observed cryo-EM density packing against the acidic patch of the motor-proximal H2A–H2B dimer. The density is in close proximity to the modeled N-terminus (residue V18) of ZNHIT1, suggesting that the density is derived from the N-terminal region of ZNHIT1 (residues 1–17). Consistently, this region is rich in positively charged residues (K4/K5/R9/R16/R17), possibly generating charge–charge interactions with the acidic patch of H2A–H2B. At the interface-2 (Fig. 3b), the helix α1 and the N-terminal region of YL1 are rich in positively charged residues (R130, K131, R134 and R147) and pack on the acidic patch of the motor-distal H2A–H2B. Thus, the two motor-associated branches individually bind to the two H2A–H2B dimers, indicating that these branches contribute to the complex positioning on target nucleosome. Furthermore, the ATPase-driven motion of the motor module may provide the driving force for destabilization of H2A–H2B and histone exchange (discussed below).

### Organization of the ARP module

The ARP module resembles an extended arm that wraps about half of the nucleosome along the DNA (Figs. 2b, 3c; Supplementary Video S1). Within the ARP module, the N-terminal region of the SRCAP subunit serves as a scaffold to organize five auxiliary subunits. The arch-shaped HSA helix spans a distance of ~170 Å along with the nucleosomal DNA. It binds the ATPase lobe1 (resembling a shoulder), a single-subunit ACTB (termed ACTB^b^, resembling an elbow), and a DMAP1–ACTL6A–ACTB^a^ heterotrimer (resembling a hand). The following linker and pre-HSA helix of the SRCAP subunit intertwine with the helical region of DMAP1, bridging the DMAP1–ACTL6A–ACTB^a^ heterotrimer and the putative YEATS4 (resembling an extended finger).

Earlier studies have shown that only one copy of ACTB associates with actin-related subunits in other ACTB-containing chromatin remodelers such as BAF/PBAF and INO80^39,45,46^ (Supplementary Fig. S5d). By contrast, locally refined cryo-EM map around ACTB^b^ at 4.2 Å (Supplementary Fig. S1d) and XLMS analysis (Supplementary Fig. S2b, c) together support the placement of an additional ACTB^b^, which binds the HSA helix spanning four turns and is positioned between the ATPase and the DMAP1–ACTL6A–ACTB^a^ (Fig. 3b). This observation agrees with the existence of the two separate actin molecules in SWR-C^15^. The SRCAP-specific ACTB^b^ may play a role in stabilizing the long HSA helix.

DMAP1, ACTL6A, and ACTB^a^ are sequentially arrayed on the HSA helix and form a folded trimer (Figs. 2b, 3c). The binding of ACTL6A–ACTB^a^ heterodimer to the HSA helix involves hydrophobic contacts and charge–charge interactions between a shallow groove of ACTL6A–ACTB^a^ and HSA helix (spanning ~12 turns of the HSA helix). DMAP1 serves as an auxiliary scaffold of the ARP module. The N-terminal β-sheet of DMAP1 makes hydrophobic interactions with the subdomain1 of ACTL6A and subdomain1 of ACTB^a^, possibly buttressing the ACTL6A–ACTB^a^ heterodimer. The SANT domain of DMAP1 binds the linker of the SRCAP subunit and the subdomain3 of ACTL6A. The following helices of DMAP1 binds the pre-HSA helix of the SRCAP subunit and the putative YEATS4. This structural observation agrees with previous studies showing that the depletion of ACTL6A ortholog (Arp4) in yeast substantially impaired the association of Swc4 (ortholog of DMAP1 in yeast)^47^.

ACTL6A–ACTB^a^ heterodimer is a shared module of the INO80-C and SRCAP-C^45,48,49^. Structural comparison with yeast Swr1^HSA^–actin–Arp4^48^ and Ino80^HSA^–Arp8–actin–Arp4^45^ subcomplexes shows similar fold of ACTL6A–ACTB/Arp4–actin (Supplementary Fig. S5d). INO80-C does not have subunits equivalent to DMAP1 and YEATS4. Complex-specific composition and organization of the ARP modules may be consistent with distinct functions of SRCAP-C (no sliding activity)^14,33^ and INO80-C (with sliding activity for nucleosome spacing)^50^.

### Multiple contacts between the ARP module and nucleosome

The ARP module makes multiple contacts with nucleosome (Fig. 3c). ACTB^b^ possesses positively charged residues (K284, R290, K291, K326, K328) that are positioned near nucleosomal DNA at SHL –4, suggesting charge–charge interactions (interface-4) (Fig. 3c). The ACTB^b^-bound HSA is rich in positively charged residues that are orientated towards nucleosomal DNA. Although no direct contact was observed in the cryo-EM map, this HSA fragment may establish contacts with DNA during SRCAP-mediated chromatin remodeling. At the interface-5 (Fig. 3c), the N-terminal loop-helix motif (residues 48–65) derived from DMAP1 contacts nucleosomal DNA at SHL 3 and helices α3 and αC of histone H2B. At the interface-6 (Fig. 3c), ACTL6A–ACTB^a^ makes no direct contact with nucleosome, whereas the ACTL6A–ACTB^a^-bound HSA fragment makes multiple contacts with nucleosomal DNA at SHL 4 through sequentially arrayed positively charged residues (R156, R159, R163, K164, R167, R171).

Around the interface-6 (Fig. 3c), weak cryo-EM map density near the motor-distal surface of the nucleosome suggests the positioning of a putative YEATS4, a YEATS domain-containing subunit that binds acylated histone H3^51–53^ (Fig. 3c, interface-7). Moreover, the locally refined cryo-EM map of ARP module shows low-resolution density between the YEATS domain and DMAP1–ACTL6A–ACTB^a^ (Supplementary Fig. S2d). XLMS analysis suggests that the density may be derived from the putative helices of YEATS4, DMAP1, and pre-HSA of the SRCAP subunit (predicted from AlphaFold^54^) (Supplementary Fig. S2b and Table S2).

The flexible positioning of YEATS4 and charge–charge interactions between nucleosomal DNA and ARP subunits suggest a non-sequence-specific binding of nucleosome. Such a mode of interaction allows the ARP module to fit the architecture of nucleosome and generate extensive interactions, which may maintain the association of SRCAP-C with nucleosome during cycles of ATP hydrolysis.

### Structure in the apo form suggests positioning of the ATPase by YL1 and the ARP module

**Fig. 4:**
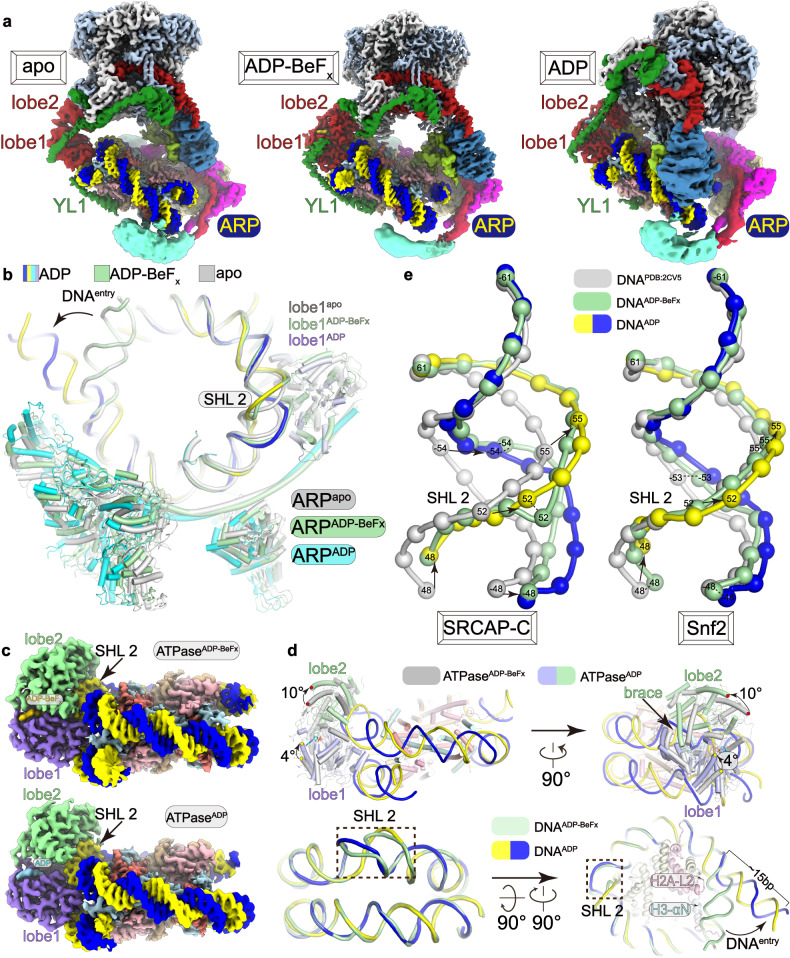
Structures in the three states reveal DNA distortion and the lack of net translocation activity. **a** Cryo-EM maps of the nucleosome-bound SRCAP-C in the apo, ADP-bound and ADP-BeF_x_-bound states, which are aligned with the nucleosome density. **b** Comparison of the ARP–nucleosome organization in the three structures. Similar gyres of DNA are omitted for clarity. **c** Close-up views of the ATPase–nucleosome in the ADP-bound and ADP-BeF_x_-bound states. **d** Superimposition of the ATPase domain (top) and nucleosomal DNA (bottom) in the two nucleotide-bound states. The red dot represents the rotation of lobe2 and the yellow dot represents the rotation of lobe1. **e** Comparison of nucleosomal DNA in the canonical nucleosome and remodeler-bound nucleosome (SRCAP-C left; Snf2 right) in the two nucleotide-bound states. Color scheme is shown and the bases of nucleosomal DNA are numbered for comparison.

Cryo-EM 3D classification of two samples (with ADP and ADP-BeF_x_) showed a shared reconstruction with characteristics of apo state (Fig. 4a; Supplementary Figs. S1c, S4a). The open conformation of the ATPase indicates the absence of nucleotide in the active site (Supplementary Fig. S5a). While the ATPase lobe1 contacts nucleosomal DNA, the lobe2 detaches from DNA. The ATPase in the apo form guides the motor module away from NCP, an organization distinct from those in the ADP-BeF_x_-bound and ADP-bound states (Fig. 4a; Supplementary Fig. S4a–c and Videos S1–S3). The ATPase is positioned near SHL 2, similar to that in the ADP-BeF_x_-bound and ADP-bound states, likely guided by the ATPase-associated YL1 and ARP module, together generating multiple contacts with the nucleosome. Despite a slight shift, YL1 and the ARP module maintain comparable nucleosome-binding modes, respectively, in the apo, ADP-bound, and ADP-BeF_x_-bound states (Fig. 4a, b). This suggests that YL1 and the ARP module maintain the association between the nucleosome and SRCAP-C, regardless of whether the ATPase is bound to nucleotide (ATP or ADP) or not.

### The ATPase-associated ARP module may prevent net DNA translocation

Locally refined cryo-EM maps at ~2.9–3.0 Å resolution supported unambiguous modeling of the ATPase and nucleosomal DNA (Fig. 4c, d; Supplementary Fig. S6 and Videos S1–S3). The ATPase in the ADP-BeF_x_-bound state adopts a fold similar to those in previously reported structures of yeast SWR-C and Snf2^14,55^ (Supplementary Fig. S5b, c). Two lobes of the ATPase together grasp nucleosomal DNA at SHL 2. A brace helix extends out of the lobe2 and bridges the two lobes. Compared to the ADP-BeF_x_-bound state, structure in the ADP-bound state shows that the ATPase is in a more relaxed conformation with the lobe1 remaining in a similar position relative to histone octamer and the lobe2 rotating ~10° towards the dyad of the nucleosome.

Compared to a canonical nucleosome, the ATPase in the ADP-bound and ADP-BeF_x_-bound states lifts the tracking strand of the nucleosomal DNA at SHL 2 from the histone surface (Fig. 4e; Supplementary Fig. S6 and Video S4). The nucleosomal DNA spanning positions 47–60 (with the entry end numbered position 1 as a reference point) exhibits distortion, and this distortion is more pronounced in the ADP-bound state. Nucleosomal DNA flanking the distorted region is almost identical in the two structures, consistent with the lack of net DNA translocation mediated by SRCAP-C^14,33^ (Fig. 4e; Supplementary Fig. S6). This structural observation differs from an early study in yeast Snf2, in which DNA distortion in the ADP-bound state propagates to the upstream region, leading to a proposed mechanism for the DNA translocation^55^ (Fig. 4e). As the ARP module binds the upstream DNA and associates with the ATPase through the HSA helix (Fig. 3b), the ARP module may restrain propagation of DNA distortion and therefore prevent net DNA translocation, a unique feature of SRCAP-C^14,33^.

### The ATPase-driven motions of the motor module

**Fig. 5:**
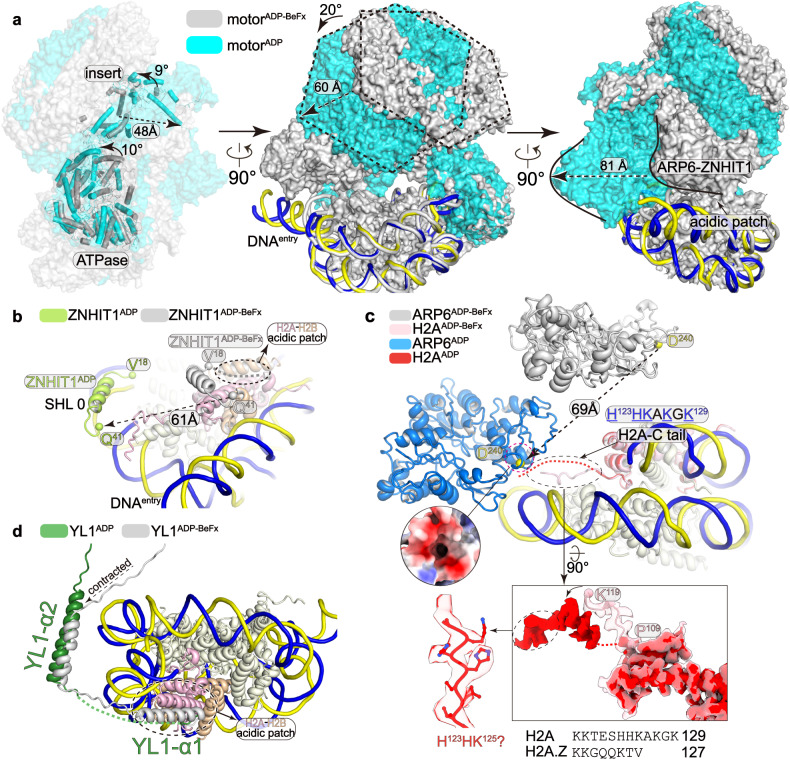
Conformational differences in the ADP-bound and ADP-BeF_x_-bound states. **a** Superimposition of the two structures with differences highlighted and indicated with arrows. Nucleosomes are superimposed for comparison. Three views showing the rotation/displacement of SRCAP, hexamer, and ARP6–ZNHIT1 dimer, respectively. **b**–**d** Close-up views of the differences in ZNHIT1 (**b**) H2A C-terminal tail (**c**) and YL1 (**d**). Conformational differences are highlighted with arrows. Structural comprision showing the dissociation of ZNHIT1 from the motor-proximal H2A–H2B (**b**). Structural comparison showing the displacement of ARP6 and the possible interaction between the relocated ARP6 and the C-terminal tail of histone H2A and the sequence alignment of the C-terminal tail of H2A and H2A.Z. The subdomain4 of ARP6 is shown in electrostatic surface and H2A tails (residues 110–127) are shown in transparent surfaces and structural models (**c**). Structural comparison showing the contraction of YL1 (**d**).

Structural comparison also suggests considerable conformational changes of the motor module, likely resulting from the changes of ATPase upon ATP hydrolysis (Figs. 4a, 5a; Supplementary Fig. S4 and Video S4). As the ATPase lobe2 undergoes rotation, the RUVBL1–RUVBL2 hexamer rotates accordingly by ~20° and displaces as far as 60 Å towards the histone octamer. The RUVBL1–RUVBL2 hexamer-associated ARP6–ZNHIT1 dimer dissociates from the motor-proximal H2A–H2B and relocates near the dyad of the nucleosome, causing a displacement of up to 81 Å. As a result, the movement of the ARP6–ZNHIT1 dimer might lead to the dissociation of the positively charged N-terminal region of ZNHIT1 from the acidic patch of H2A–H2B (Fig. 5b). The entry DNA undergoes unwrapping from the histone octamer by ~15 bp, which would otherwise cause a steric clash with the relocated ARP6–ZNHIT1. Nucleosomal DNA detaches from H3 (helix αN) at SHL 6.5 and from H2A (loop L2) at SHL 5.5, resulting in a partially exposed and possibly destabilized H2A–H2B dimer (Fig. 4d).

In the ADP-BeF_x_-bound state, the C-terminal tail of histone H2A (residues 110–119) interacts with the αN helix of H3, and the C-terminal basic residues are positioned near the entry DNA (Fig. 4d; Supplementary Video S4). Consistent with the entry DNA detachment, the cryo-EM map in the ADP-bound state showed the absence of the H2A C-terminal tail and the presence of density extending out of the H2A core and winding over the subdomain4 of ARP6 (Fig. 5c). It suggests that the density is derived from the H2A tail (residues 110–127) and the basic motif (H^123^HKAKGK^129^, positively charged residues are underlined) may generate charge–charge interactions with the acidic pocket of ARP6. Sequence alignment showed that H2A.Z lacks equivalent basic residues (G^122^QQKTV^127^), suggesting that H2A.Z may differ from H2A in binding ARP6 (Supplementary Fig. S3g). The rotation of the motor module also induces a contraction of YL1 on the ATPase-bound linker and the helix α1 on the motor-distal H2A–H2B, with the latter being flexible and only a short fragment (residues 130–138) associated with H2A–H2B (Fig. 5d).

The above structural comparison suggests that the ATPase-driven motions of the motor module may provide a driving force for the destabilization and eviction of H2A–H2B dimer. Transient motions of H2A–H2B during histone exchange^31^ may not be captured by cryo-EM structure determination of the complexes in static states. Eviction of H2A–H2B may also require histone-binding subunits, histone chaperons, and H2A.Z–H2B dimer for replacement, which were not present in the cryo-EM samples.

### Acute degradation reveals the crucial role of ZNHIT1 in histone H2A-H2A.Z exchange in cells

**Fig. 6:**
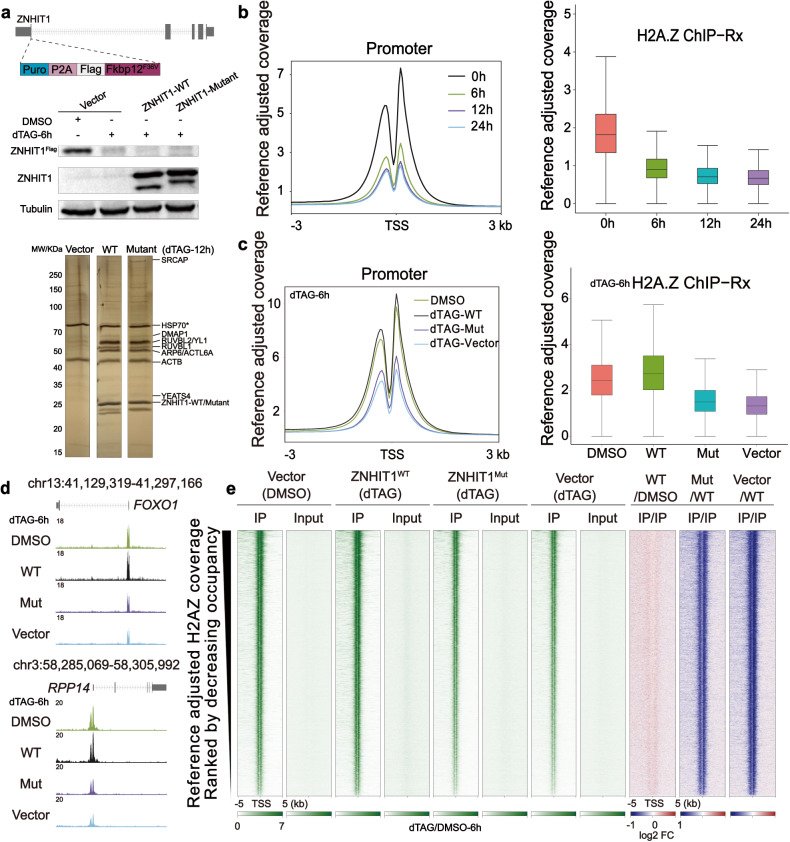
The H2A-binding region of ZNHIT1 is required for histone exchange in cells. **a** Schematic diagram of establishing ZNHIT1-dTAG DLD-1 cells and validation of ZNHIT1 degradation and rescue. An empty vector, WT and mutant ZNHIT1 were transfected into the ZNHIT1-dTAG cells, respectively. The expression of target proteins was detected by western blot. Complex composition was validated by silver staining of the SRCAP-C purified from the indicated ZNHIT1-dTAG cells. **b** Metaplot and boxplot representation of H2A.Z occupancy in ZNHIT1-dTAG cells treated with DMSO or dTAG for the indicated times. **c** Metaplot and boxplot representation of H2A.Z occupancy in DMSO/dTAG-treated ZNHIT1-dTAG cells with overexpression of WT or mutant ZNHIT1. **d** Representative track examples showing H2A.Z occupancy in DMSO/dTAG-treated ZNHIT1-dTAG cells with overexpression of WT or mutant ZNHIT1. **e** Heatmaps of H2A.Z occupancy centered at TSSs of promoters ranked by decreasing occupancy in DMSO/dTAG-treated ZNHIT1-dTAG cells with overexpression of WT or mutant ZNHIT1.

Among all the nucleosome-binding subunits, ZNHIT1 directly contacts H2A–H2B and seems to pull H2A–H2B out of nucleosome for dimer exchange (Figs. 3b, 5b). To investigate whether ZNHIT1 is essential for H2A.Z incorporation in cells, we used the degradation tag (dTAG) system^56^ by integrating the Flag-FKBP12^F36V^ tag at the N-terminus of the endogenous ZNHIT1 (ZNHIT1-dTAG) in DLD-1 cells (Fig. 6a). The level of histone H2A.Z was detected using chromatin immunoprecipitation followed by sequencing with reference exogenous genome (ChIP-Rx). The addition of dTAG-13 led to a time-dependent decrease in ZNHIT1 protein level, which reached the plateau after 12 h of treatment (Supplementary Fig. S7a). By contrast, the degradation of ZNHIT1 did not impact the stability of the other two representative SRCAP-C subunits, YL1 and YEATS4. We next performed H2A.Z ChIP-Rx on ZNHIT1-dTAG cells treated with dTAG-13 for 6 h, 12 h, and 24 h, respectively. Genome-wide analysis indicated the enrichment of H2A.Z in transcription start site (TSS) regions (Fig. 6b; Supplementary Fig. S7c), consistent with the observation in previous studies^19,57^. ChIP-Rx data at three time points showed that the occupancy of H2A.Z on the genome exhibited an evident decrease by 6-h treatment and a dramatic decrease by 24-h treatment (Fig. 6b; Supplementary Fig. S7b, c). SRCAP-C seems to be consistently required for H2A.Z occupancy on promoters, whereas INO80-C may lead to H2A.Z–H2B replacement by H2A–H2B^4,9^. These results demonstrate that ZNHIT1 is essential for global H2A.Z deposition, in line with previous studies in mice^58,59^.

The contacts between the basic residues of the N-terminal region of ZNHIT1 and the H2A–H2B acidic patch in the ADP-BeF_x_-bound state suggest a key role of these residues in H2A-H2A.Z exchange (Fig. 3b). We next generated rescue cell lines by overexpressing WT or a charge-reversal mutant ZNHIT1 (K4E, K5E, R9E, R16E, R17E) in ZNHIT1-dTAG cells with rapid degradation of endogenous ZNHIT1 protein (Fig. 6a). Immunoprecipitation of FLAG-tagged WT and mutant ZNHIT1 from the two rescue cell lines showed comparable complex composition, suggesting that the ZNHIT1 mutation did not disrupt the SRCAP-C complex. Comparison of H2A.Z ChIP-Rx in dTAG-13-treated cells with overexpression of WT ZNHIT1 and an empty pLVX vector showed that WT ZNHIT1, but not the empty vector, fully restored the levels of H2A.Z (Fig. 6c–e). By contrast, the ZNHIT1 mutant failed to rescue the dramatic decrease in H2A.Z level. The change of H2A.Z levels shown in heatmaps indicated that WT but not ZNHIT1 mutant restrained H2A.Z occupancy at genome-wide levels. These results suggest that the N-terminal region of ZNHIT1 is required for H2A-H2A.Z exchange in cells. Consistently, an earlier study showed that mutation of the H2A helix α2, which directly contacts the N-terminal loop of ZNHIT1, largely decreased in vitro histone exchange activity of SWR-C^33^.

### A working model of histone exchange

**Fig. 7:**
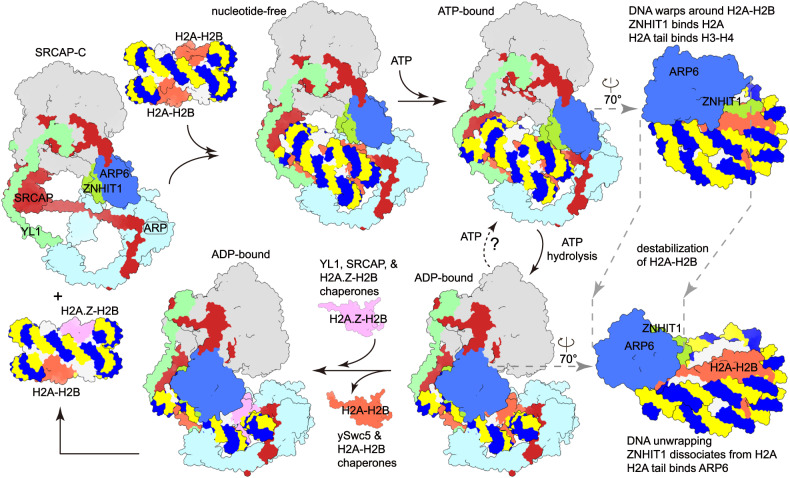
A working model for histone exchange by SRCAP-C. Cartoons were generated from cryo-EM maps. Right panels show close-up views of the nucleosome-bound ARP6–ZNHIT1 with other portions omitted for clarity. Multiple cycles of ATP hydrolysis may be required to destabilize H2A–H2B, followed by a successful histone exchange. Histone chaperones and associated H2A.Z–H2B may also facilitate histone exchange. One H2A–H2B is replaced in each exchange reaction.

We here proposed a working model for H2A-H2A.Z exchange (Fig. 7). SRCAP-C binds the H2A-containing nucleosome through the motor and ARP modules and the binding of ATP leads to a transition from the apo state to the ATP-bound state (Supplementary Video S5). In the ATP-bound state, ZNHIT1 binds the acidic patch of the motor-proximal H2A–H2B dimer, entry DNA wraps around the H2A–H2B dimer, and the C-terminal tail of H2A associates with the H3–H4 dimer and entry DNA. Upon ATP hydrolysis, the ATPase undergoes conformational changes relative to the nucleosome and the lobe2-associated RUVBL1–RUVBL2 hexamer transduces the motion of the ATPase to the ARP6–ZNHIT1 dimer (Supplementary Video S4). As a result, ARP6–ZNHIT1 moves away from the H2A–H2B surface to the dyad of the nucleosome leading to the unwrapping of the entry DNA by ~1.5 turns, which may destabilize H2A–H2B. The motion of ARP6–ZNHIT1 may further destabilize H2A–H2B through two contacts. The N-terminal H2A-binding motif of ZNHIT1 tends to pull H2A–H2B out of nucleosome. By binding the relocated ARP6, the C-terminal tail of H2A dissociates from the entry DNA and H3–H4 dimer, which may also contribute to H2A–H2B destabilization. As H2A.Z differs from H2A at their C-terminal tails, H2A.Z may be less prone to destablization. Other differences between H2A and H2A.Z might also play a role in the selective replacement of H2A with H2A.Z, rather than the reverse reaction.

## Discussion

### Structure comparison of SRCAP-C and SWR-C suggests distinct mechanisms

In an earlier structural study^14^, while the 14-subunit SWR-C was used for structure determination, four yeast-specific subunits and the 6-subunit ARP module were not observed (Supplementary Fig. S3a). The structure and function of these subunits require further investigation and the mechanistic understanding of SWR-C is further hampered by the lack of structure in the ADP-bound state. Nevertheless, structures of SRCAP-C and SWR-C in the ADP-BeF_x_-bound state show that the motor modules similarly bind to nucleosome. However, structure comparison did reveal the following differences.

SWR-C–nucleosome structure in the ADP-BeF_x_-bound state^14^ showed that the histone core “flexes” and the upper tier, comprising a histone dimer and part of the H3–H4 tetramer, twists relative to the lower tier (Supplementary Fig. S3e). The entry DNA is unwrapped and the unwrapping requires ATP binding but not ATP hydrolysis. Yeast Swc6 (ZNHIT1 homolog) binds the unwrapped portion of the entry DNA and binds H2A on helix αC (Supplementary Fig. S3a, c). By contrast, in SRCAP-C–nucleosome structures (ADP- and ADP-BeF_x_-bound states), histone octamer remains in canonical conformation and DNA unwrapping was only observed in the ADP-bound state, in which ZNHIT1 binds neither the entry DNA nor the H2A–H2B dimer (Fig. 5b; Supplementary Fig. S3b). Consistently, sequence alignment shows low homology in the nucleosome-binding regions of the human ZNHIT1 and yeast Swc6 subunits (Supplementary Fig. S3f). Besides, we assembled the SRCAP-C–NCP in the presence of purified H2A.Z–H2B dimer under a condition similar to assembly of SWR-C–NCP^14^. The cryo-EM map is almost identical to that of SRCAP-C–NCP without H2A.Z–H2B (Supplementary Fig. S3d). These analyses suggest that the destabilization of H2A–H2B dimer by SRCAP-C and SWR-C may not be in precisely the same manner.

### The DNA unwrapping and histone exchange

The structures and the proposed model in this work agree with earlier single-molecule analyses. For example, SWR-C unwraps nucleosomal DNA and removes H2A–H2B from the same face of the nucleosome^31,60^. The assays showed that SWR-C only unwraps DNA on the same nucleosome face as the H2A–H2B dimer is displaced, leaving the nucleosomal DNA on the opposite face unperturbed. Consistently, an earlier biochemical study showed the replacement of one H2A.Z–H2B dimer at a time^40^. SWR-C induces the productive unwrapping of nucleosomal DNA correlated with histone exchange^31^. The duration of these unwrapping events, in the scale of seconds, is significantly shorter than the binding lifetime of the SWR-C–NCP complex, which extends into the scale of minutes. Considering the time scale of ATP hydrolysis, this observation suggests that the nucleosome bound by SWR-C primarily maintains its conventional wrapping and that the transition from the ATP-bound state (wrapped DNA) to the ADP-bound state (unwrapped DNA) may correlate with DNA unwrapping and histone exchange in a short duration. Structural and single-molecule analyses together support the proposed functional correlation between conformational changes of SRCAP-C, DNA unwrapping, and histone exchange.

Previous studies have also shown that histone exchange reactions are influenced by asymmetric extranucleosomal DNA lengths, resulting in a bias towards the nucleosome side distal to the longer extranucleosomal DNA^31,32^. However, others did not observe the preference in exchange of histone dimer distal to the longer extranucleosomal DNA and proposed that the exchanged dimer is proximal to the ATPase-positioned SHL 2, independent of which side any extranucleosomal DNA might be attached to^14,61^. Consistent with the latter model, our SRCAP-C–NCP structures show that the ATPase binds nucleosome (108N12) at SHL 2, the longer extranucleosomal DNA is unwrapped, and its proximal H2A–H2B dimer tends to be destabilized. Nevertheless, two H2A–H2B dimers of one nucleosome could be replaced by H2A.Z–H2B dimers through two rounds of reaction, suggesting that such preference, if exists, may not be a major regulatory factor.

### Histone exchange by SRCAP-C may require other factors

No histone exchange activity was detected in our experimental conditions using the purified recombinant SRCAP-C (data not shown). We suspected that the 10-subunit human SRCAP-C, despite the presence of all currently characterized components, is highly purified and may lack some unknown factor(s) required for histone exchange activity. Earlier studies reported histone exchange evidenced by in vitro assays using yeast SWR-C^8,15,16,32,62^ and human SRCAP-containing complexes^11,63^, the latter of which may contain some undefined subunits/factors essential for histone exchange.

The purified SRCAP-C in this study lacks subunits equivalent to Swc3, Swc5, Swc7, and Bdf1, four subunits unique to the yeast SWR-C. Studies reveal that among the 14 distinct subunits of SWR-C, at least seven (Swr1, the catalytic subunit, and accessory subunits Swc2, Arp6, Swc6, Swc5, Arp4, and Yaf9) are necessary for the core histone replacement reaction in vitro^47,64^. The Swc5 subunit, no equivalent in human SRCAP-C, is indispensable for H2A.Z replacement in yeast both in vitro and in vivo^38,64–66^. Swc5 is required for SWR-C ATPase stimulation, suggesting that Swc5 is required to couple substrate recognition to ATPase activation. In vitro studies showed that the acidic N-terminus of Swc5 preferentially binds to the H2A–H2B dimer, and thus it may function as histone chaperone to assist H2A ejection when H2A.Z is inserted into the nucleosome.

To complete a histone exchange, the destabilized H2A–H2B has to be replaced by H2A.Z–H2B, a process might require histone chaperones Nap1^67^, HIRA^18^ and Chz1^16,17^, and/or the H2A/H2A.Z-binding domains/motifs within YL1^63,64,68,69^, SRCAP^47,62^, and Swc5^38^. For example, Chz1 is a specific chaperone for the histone variant H2A.Z in budding yeast. The ternary complex formed by Chz1 and H2A.Z–H2B dimer is the major substrate of SWR-C in yeast cells^17^. Furthermore, Chz1 facilitates SWR-C-mediated H2A.Z deposition by alleviating inhibition caused by aggregation of excess free histones^16^. YL1^63,69^ and Swr1-Z domain^47,62^ specifically recognize H2A.Z–H2B dimer and may deliver the H2A.Z–H2B dimer to assemble a nucleosome. HIRA complex collaborates with SRCAP-C to deposit H2A.Z onto the promoters in mouse embryonic stem cells (mESCs), which could interact with SRCAP-C through the Hira subunit as verified by in vivo and in vitro biochemical assays^18^. Nap1, an H2A–H2B histone chaperone^67^, has been shown to remove the H2A–H2B dimer from SWR-C after its displacement from the nucleosome^31^.

## Materials and methods

### Antibodies and cell culture

Antibodies were as follows: H2A.Z (ab4174, abcam), ZNHIT1 (16595-1-AP, Proteintech), VPS72 (15143-1-AP, Proteintech), YEATS4 (A6318, ABclonal), Flag (SLAB0101, Smart-Lifesciences), tubulin (AC008, Abclonal). 293T, DLD-1, and mouse embryonic fibroblast (MEF) cells were grown in Dulbecco’s minimum essential medium (DMEM) (Gibco) supplemented with 10% fetal bovine serum (FBS) (Yeasen). HEK Expi293 cells were grown in suspension in Union 293 Medium (Union) at 37 °C, 5% CO_2_, 130 rpm.

### SRCAP expression and purification

The ten full-length ORFs of SRCAP-C subunits were subcloned into modified pMLink vector. SRCAP and VPS72 were tagged with an N-terminal Flag, 4× Protein A, followed by an HRV-3C cleavage site. All human SRCAP-C subunits were co-transfected into suspension Expi293F cells using polyethylenimine (Polysciences). Cells were cultured for 72 h at 37 °C and harvested by centrifugation. For complex purification, all the steps were performed at 4 °C. Cells were disrupted in lysis buffer containing 50 mM HEPES (pH 8.0), 300 mM NaCl, 5% (v/v) glycerol, 0.2% (w/v) CHAPS, 2 mM MgCl_2_, 0.5 mM EDTA, 2 mM DTT, 1 mM phenylmethylsulfonyl fluoride (PMSF), 1 μg/mL Aprotinin, 1 μg/mL Pepstatin, 1 μg/mL Leupeptin for 30 min. Raw cell lysate was clarified by centrifugation at 38,420× *g* for 30 min. The supernatant was incubated with IgG resin for 4 h and washed thoroughly with wash buffer containing 20 mM HEPES (pH 8.0), 300 mM NaCl, 5% (v/v) glycerol, 2 mM MgCl_2_, 2 mM DTT. After on-column digestion overnight, immobilized protein was eluted using wash buffer with the NaCl concentration adjusted to 150 mM, and then further purified through ion-exchange chromatography (Mono Q 5/50 GL column, GE Healthcare). Peak fractions were collected and concentrated to ~3 mg/mL and used for subsequent SRCAP-C–NCP assembly or flash-freezing in liquid nitrogen in small aliquots for biochemical analyses.

### Preparation and acetylation of nucleosomes

Canonical human octamer and DNA fragments were prepared as described previously^39^. DNA fragments for nucleosome reconstitution were prepared by PCR amplification. Nucleosome reconstitution was performed by mixing DNA with octamer at an equimolar ratio, with a linear salt gradient dialysis according to a previously published protocol^46^. Nucleosomes were dialyzed to 1× HE buffer containing 10 mM HEPES (pH 8.0) and 0.1 mM EDTA. Acetylation of nucleosome was performed as described previously^39^. Briefly, nucleosome and HATs were first incubated in 1:1 stoichiometry in reaction buffer containing 50 mM Tris-HCl (pH 8.0), 0.1 mM EDTA, 10% (v/v) glycerol, 1 mM DTT, 1 mM PMSF at 30 °C for 5 min, followed by the addition of 50 μM acetyl-CoA for another 30 min at 30 °C.

The nucleosome was assembled for ATPase assay and then acetylated for cryo-EM. The DNA sequence is as follows (the ‘601’ positioning sequence is underlined):

ACTGGCACCGGTTTAAACGCTGTTCAATACATGCCCGGCACCCCCCCTCGAGGTCGACGGTATCGATAAGCTTGATATCCTCGGGACCCAAGCGACACCGGCACTGGAACAGGATGTATATATGTGACACGTGCCTGGAGACTAGGGAGTAATCCCCTTGGCGGTTAAAACGCGGGGGACAGCGCGTACGTGCGTTTAAGCGGTGCTAGAGCTGTCTACGACCAATTGAGCGGCCTCGGCACCGGGATTCTCCAGGGAATTCCCCAG

### In vitro ATPase assay

The ATPase activity was performed with the ADP-Glo™ Kinase Assay Kit following the manufacturer’s instructions (V6930, Promega). In brief, 200 nM nucleosome was mixed with 200 nM SRCAP complex or PBAF complex^39^ in buffer containing 20 mM HEPES (pH 8.0), 100 mM NaCl, 2 mM MgCl_2_, 1 mM DTT, 5% (v/v) glycerol, 0.1 mg/mL BSA, for reaction (5-µL volume). The reactions were started with the addition of 0.1 mM ATP at 30 °C for 30 min and stopped by adding ADP-Glo^TM^ Reagent.

### Complex assembly and cryo-EM sample preparation

For complex assembly, the purified SRCAP complex was mixed with acetylated NCP at a ratio of 1:1, followed by incubation with 1 mM ATP for 30 min at 30 °C and then with 0.5 mM ADP (SRCAP-C–NCP^ADP^) or 0.5 mM ADP-BeF_x_ (0.5 mM ADP, 7 mM NaF, and 1 mM BeSO_4_) (SRCAP-C–NCP^ADP-BeFx^) for 15 min at 30 °C. The complexes were subjected to gradient fixation^70^. In brief, each sample was loaded onto a gradient generated from a glycerol light solution containing 15% (v/v) glycerol, 20 mM HEPES (pH 8.0), 50 mM NaCl, 2 mM MgCl_2_, 2 mM DTT and a glycerol heavy solution containing 35% (v/v) glycerol, 20 mM HEPES (pH 8.0), 50 mM NaCl, 2 mM MgCl_2_, 2 mM DTT and 0.01% (v/v) glutaraldehyde. Centrifugation was performed for 15 h at 274,400× *g* in an SW41Ti swinging bucket rotor (Beckman) at 4 °C. Peak fractions were pooled and quenched with 100 mM Tris-HCl (pH 8.0). The crosslinked SRCAP-C–NCP complex was concentrated and dialyzed overnight against a buffer containing 20 mM HEPES (pH 8.0), 50 mM NaCl, 2 mM MgCl_2_, 2 mM DTT. The two Grafix solutions and dialysis buffer contain 0.5 mM ADP (SRCAP-C–NCP^ADP^) or 0.5 mM ADP-BeF_x_ (SRCAP-C–NCP^ADP-BeFx^).

For negative staining EM grid preparation, 5 µL of SRCAP-C–NCP sample were applied onto glow-discharged copper grids supported by a continuous thin layer of carbon film for 60 s before negative staining by 2% (w/v) uranyl acetate solution at room temperature. The grids were prepared in the Ar/O_2_ mixture for 15 s using a Gatan 950 Solarus plasma cleaning system with a power of 35 W. The negatively stained grids were loaded onto a Thermo Fisher Scientific Talos L120C microscope equipped with a Ceta CCD camera and operated at 120 kV at a nominal magnification of 92,000× , corresponding to a pixel size of 1.58 Å on the specimen.

For cryo-EM grid preparation, samples (4 μL at a concentration of ~0.6 mg/mL) were applied to freshly glow-discharged Quantifoil R1.2/1.3 holey carbon grids. After incubation for 5 s at 4 °C and 100% humidity, the grids were blotted for 1 s with blot force of –2 in a Thermo Fisher Scientific Vitrobot Mark IV and plunge-frozen in liquid ethane at liquid nitrogen temperature. The grids were prepared in the H_2_/O_2_ mixture for 60 s using a Gatan 950 Solarus plasma cleaning system with a power of 5 W. The ø 55/20 mm blotting paper from TED PELLA was used for plunge freezing.

### Cryo-EM data collection

The cryo-EM grids were loaded onto a Thermo Fisher Scientific Titan Krios transmission electron microscope operated at 300 kV for data collection. Cryo-EM images were automatically recorded by a post-GIF Gatan K3 Summit direct electron detector in the super-resolution counting mode using Serial-EM with a nominal magnification of 64,000× in the EFTEM mode, which yielded a super-resolution pixel size of 0.667 Å on the image plane, and with defocus values ranging from 1.0 μm to 2.5 μm. Each micrograph stack was dose-fractionated to 40 frames with a total electron dose of ~50 e^–^/Å^2^ and a total exposure time of 3.6 s. 11,365 micrographs of SRCAP-C–NCP^ADP-BeFx^ and 6027 micrographs of SRCAP-C–NCP^ADP^ were collected for further processing.

### Image processing

Drift and beam-induced motion correction were applied to the super-resolution movie stacks using MotionCor^71^ and binned 2-fold to a calibrated pixel size of 1.334 Å/pix. The defocus values were estimated by Gctf^72^ from summed images without dose weighting. Other procedures of cryo-EM data processing were performed in RELION v3.1^73,74^ using the dose-weighted micrographs.

For data processing of SRCAP-C–NCP^ADP-BeFx^, 6,105,534 particles were picked by automatic particle picking with reference and subjected to reference-free 2D classification. Particles were selected from good 2D classes for the initial 3D classification, using a 60 Å low-pass filtered initial model from the cryo-EM map of SWR-C–NCP (EMD-4395). 2,603,327 particles were selected from good 2D and 3D classes for further 3D classification. Then 475,617 particles in ADP-BeF_x_-bound state were selected from a good 3D class, which were used for refinement, yielding a reconstruction of SRCAP-C–NCP^ADP-BeFx^ complex at 3.3 Å resolution, masked ATPase–NCP^ADP-BeFx^ at 3.1 Å resolution. 1,092,654 particles in the nucleotide-free state were selected for refinement, yielding a reconstruction of SRCAP-C–NCP^apo^ complex at 3.3 Å resolution, masked ATPase–NCP^apo^ at 3.4 Å resolution. As the ARP module adopts almost identical conformation in the different nucleotide-bound states, we combined the particles of apo and ADP-BeF_x_-bound state to obtain a reconstruction to a higher resolution. 1,568,271 particles of two conformations were used to subtract the ARP module, followed by 3D classification. 192,819 particles were selected from a good 3D class, which were used for refinement, yielding a reconstruction of the ARP module at 3.3 Å resolution.

For data processing of SRCAP-C–NCP^ADP^, 3,463,395 particles were picked by automatic particle picking with reference and subjected to reference-free 2D classification. Particles were selected from good 2D classes for the initial 3D classification, using a 60 Å low-pass filtered initial model from the cryo-EM map of SWR-C–NCP (EMD-4395). 1,535,342 particles were selected from good 2D and 3D classes for further 3D classification. 555,303 particles of the complex in ADP-bound state were selected from good 3D classes, which were used for refinement, yielding a reconstruction of SRCAP-C–NCP^ADP^ complex at 3.3 Å resolution, masked ATPase–NCP^ADP^ at 3.1 Å resolution. These particles were used to subtract ACTB^b^ and were subjected to 3D classification. 54,761 particles were selected from good 3D classes and were used for refinement, yielding a reconstruction of ACTB^b^ at 4.2 Å resolution.

All reported resolutions are calculated based on the gold-standard Fourier shell correlation (FSC = 0.143) criterion. The GSFSC curves were corrected for the effects of a soft mask with high-resolution noise substitution. All cryo-EM maps were sharpened by applying a negative B-factor estimation in RELION v3.1. All the visualization and evaluation of the 3D volume map were performed with UCSF Chimera or UCSF ChimeraX^75^, and the local resolution variations were calculated using RELION v3.1.

### Model building and structure refinement

The overall cryo-EM maps and locally refined maps of SRCAP-C–NCP in the apo, ADP-bound, and ADP-BeF_x_-bound states were used for model building. The structures of SRCAP complex (PDB: 6IGM)^37^ and nucleosome (PDB: 2CV5)^76^, and the structures of YL1 (Identifier: AF-Q15906-F1), ZNHIT1 (Identifier: AF-O43257-F1), DMAP1 (Identifier: AF-Q9NPF5-F1), YEATS4 (Identifier: AF-O95619-F1), ACTL6A (Identifier: AF-O96019-F1), and ACTB (Identifier: AF-P60709-F1) from AlphaFold2^54^ were used as initial structural references, which were fitted into the density maps using UCSF Chimera. The structural model was then manually adjusted in COOT^77^ according to the density map. At the current resolution, the density map of DNA phosphate groups is clear to be positioned easily. Hence, we first determined the phosphate backbone of dsDNA. Since the sizes of base density are different, we can distinguish between pyrimidine (T or C) and purine (A or G) clearly. The final model refinement was carried out using Phenix^78^ with secondary structure and geometry restraints with default parameters followed by further manual adjustment in COOT to achieve models with good stereochemistry evaluated using MolProbity^79^. Statistics of the map reconstruction and model refinement can be found in Supplementary Table S1. Map and model representations in the figures and movies were prepared by PyMOL (https://pymol.org/), UCSF Chimera, or UCSF ChimeraX.

### Generating dTAG and rescues cell lines

To generate ZNHIT1-dTAG cells by the endogenous knock-in, Precise Integration into Target Chromosome (PITCh) sgRNA/Cas9 and donor plasmids were mixed with 1 × 10^6^ DLD-1 cells followed by electroporation^56^. After recovering for 2 days without antibiotic selection, cells were serially diluted and cultured with 1 mg/mL puromycin (Meilunbio) for 10–14 days. Single-clone colonies were picked, expanded, and genotyped by genomic DNA PCR targeting the integration site. For homogeneous knock-in clones, protein degradation efficiency was verified by DMSO and dTAG-13 treatment for 3 h followed by western blotting.

To generate rescue cell lines, ZNHIT1-dTAG cells were initially infected with lentivirus expressing WT ZNHIT1, mutant ZNHIT1(K4E, K5E, R9E, R16E, R17E) cloned into pLVX vector with blasticidin resistance gene, and then selected with antibodies for 2 weeks.

### Immunoprecipitation

Rescue cell lines of WT ZNHIT1, mutant ZNHIT1, and empty pLVX vector were treated with dTAG-13 for 12 h, and then disrupted using lysis buffer mentioned above for 30 min. After centrifugation at 17,000× *g* for 15 min, the supernatant was collected to incubate with Flag-M2 beads (Sigma) for 4 h. After immunoprecipitation, protein was washed three times with wash buffer mentioned above and then eluted using wash buffer containing 400 ng/μL Flag peptide (Meilunbio) additionally. All the steps were performed at 4 °C. Samples were subject to SDS-PAGE.

### ChIP-Rx and data analysis

ChIP-Rx was performed as described previously^80^. For each ChIP assay, 1 × 10^7^ DLD-1 cells were used as described, and 1 × 10^6^ MEF cells were mixed with DLD-1 cells as the spike-in. The mixed supernatant was incubated with 3 µg antibody overnight. The libraries were prepared with the VAHTS Universal Plus DNA Library Prep Kit for Illumina (Vazyme).

The raw paired-end ChIP-Rx reads were trimmed by Trim Galore v0.6.7 (https://www.bioinformatics.babraham.ac.uk/projects/trim_galore/) to remove adapters and low-quality sequences (-q 25 -e 0.1 --stringency 4) and then aligned to the human genome hg19 and mouse genome mm10 assembly using Bowtie v2.5.0^81^ with default parameters. Unmapped reads, low mapping quality reads (MAPQ < 30), and PCR duplicates were filtered out by using SAMtools v1.16.1^82^ (parameters: -F 3844 -f 2 -q 30) and Picard v2.27.5 with default parameters (https://broadinstitute.github.io/Picard/). Reads mapping to mm10 were computed using SAMtools flagstat^82^, and the normalization factor was determined as 1e6/mm10_count. Normalized bigwig files were generated by deepTools v3.5.1^83^. Any reads mapping to ENCODE blacklist regions were excluded^84^ using BEDTools v2.30.0^85^. Peaks were called using macs2 v2.2.7.1^86^ with q-value threshold of 0.05.

TSSs in DLD-1 cells were determined using previously published PRO-cap data^87^. Genome annotation and reference genome sequences were downloaded from the UCSC Genome Browser. RefSeq-validated transcripts with PRO-cap signal at the region between –10 bp and +300 bp of TSS were selected. For protein-coding transcripts with multiple isoforms, the transcript with the most significant PRO-cap signal was selected as the representative gene and then TSS was defined by the position of the maximum PRO-cap signal. Finally, transcripts within ±1 kb of the nearest gene were removed.

### XLMS analysis

The XLMS analysis was performed as previously described^39^. The purified SRCAP complex (0.5 μM) was incubated with nucleosome at a ratio of 1:1 in the presence of ADP-BeF_x_ or ADP followed by crosslinking MS analyses. The SRCAP-C–NCP complex was incubated with DSS (1.25 mM) at 25 °C with shaking at 500 rpm (ThermoMixer) for 1 h. Reaction was terminated by adding 20 mM ammonium bicarbonate (Sigma). The crosslinked sample was precipitated with cooled acetone and dried in a speed vac. The pellet was dissolved in 8 M urea, 100 mM Tris-HCl (pH 8.5), followed by TCEP reduction, iodoacetamide (Sigma) alkylation, and trypsin (Promega) digestion overnight at 37 °C using a protein/enzyme ratio of 50:1 (w/w). Tryptic peptides were desalted with Pierce C18 spin column (GL Sciences) and separated in a proxeon EASY-nLC liquid chromatography system by applying a step-wise gradient of 0–85% acetonitrile (can) in 0.1% foricacid. Peptides eluted from the liquid chromatography were directly electrosprayed into the mass spectrometer with a distal 2 kV spray voltage. Data-dependent tandem mass spectrometry (MS/MS) analyses were performed on Thermo Q-Exactive instrument in a 60-min gradient. The acquired raw data files were processed with pLink2 software^88^ and the results were visualized using the xiNET^89^.

### Supplementary information

Supplementary information

Supplementary Table S2

Supplementary Video S1

Supplementary Video S2

Supplementary Video S3

Supplementary Video S4

Supplementary Video S5

## Data Availability

The cryo-EM maps and coordinates have been deposited to the Electron Microscopy Data Bank (EMDB) and Protein Data Bank (PDB) with accession codes of EMD-37988 and 8X19 (PDB) (ADP-BeF_x_-bound state), EMD-37990 and 8X1C (PDB) (ADP-bound state), and EMD-37984 and 8X15 (PDB) (apo state). Sequencing data have been deposited at the Gene Expression Omnibus (GEO) under accession number GSE249825.
