# Supplementary material for: Structural insights into histone exchange by human SRCAP complex
Source: Cell Discov. 2024 Feb 8;10:15. doi: 10.1038/s41421-023-00640-1 (PMC10853557; doi:10.1038/s41421-023-00640-1)
Supplement: Supplementary file 1 — Supplementary information [file 41421_2023_640_MOESM1_ESM.pdf]

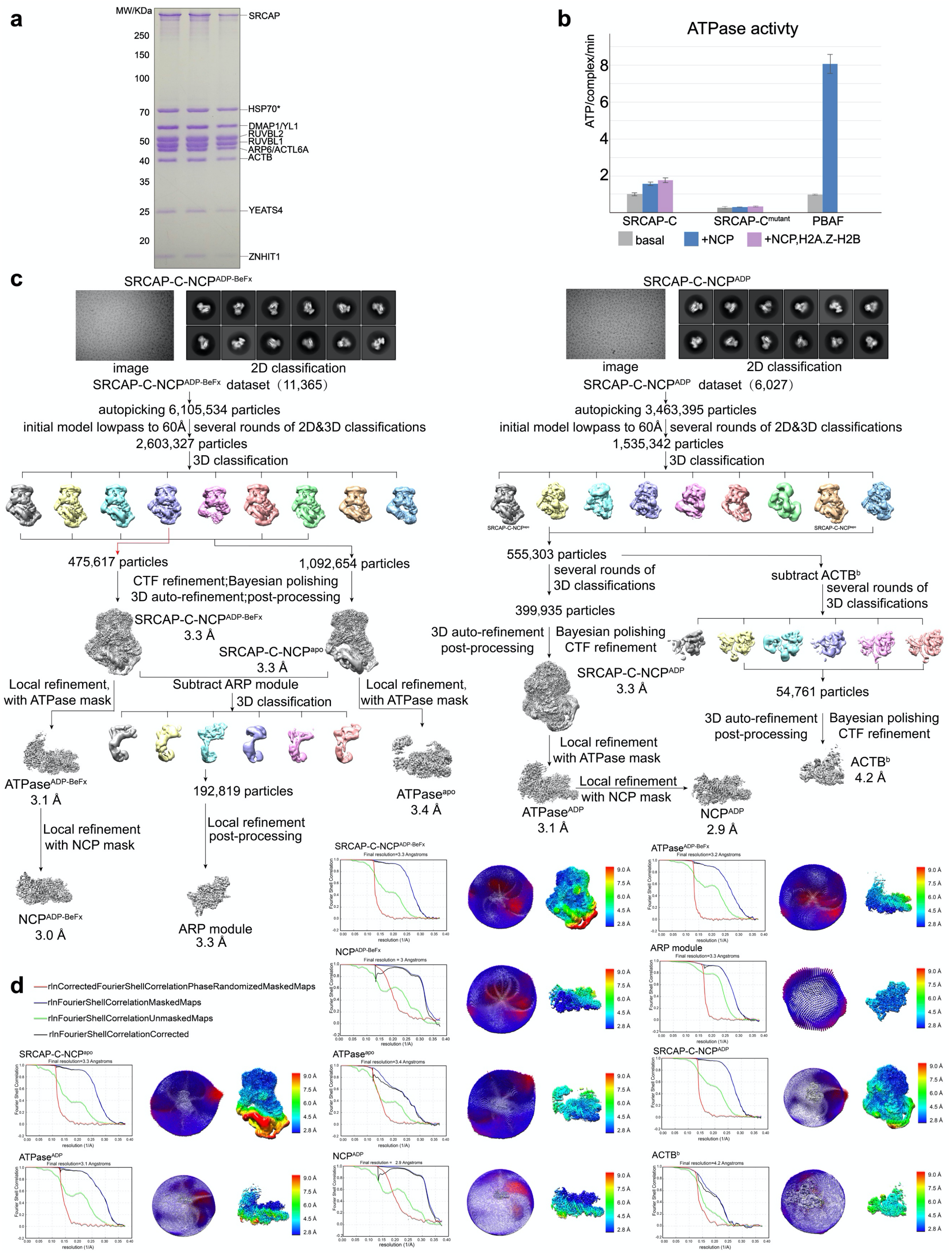

Supplementary Fig. S1: Protein purification and cryo-EM data processing.

a Peak fractions of ion exchange chromatography were subjected to SDS-PAGE and visualized by

Coomassie blue staining. * represents a commonly co-purified contaminant chaperone protein HSP70. **b** ATPase activities of SRCAP-C, SRCAP-C containing a SRCAP ATPase-dead mutant (K649G, R2151G, R2154G), and a positive control PBAF. The experiments were performed in triplicate. **c** Date collection and processing of the nucleosome-bound SRCAP-C in the presence of ADP (right) and ADP-BeF_x (left), respectively. Cryo-EM micrograph and 2D classification (top) and flow charts of the data processing (bottom). **d** Angular distributions, local resolution estimation, and histogram of directional FSC plots of the cryo-EM maps of global refinement and focused refinements.

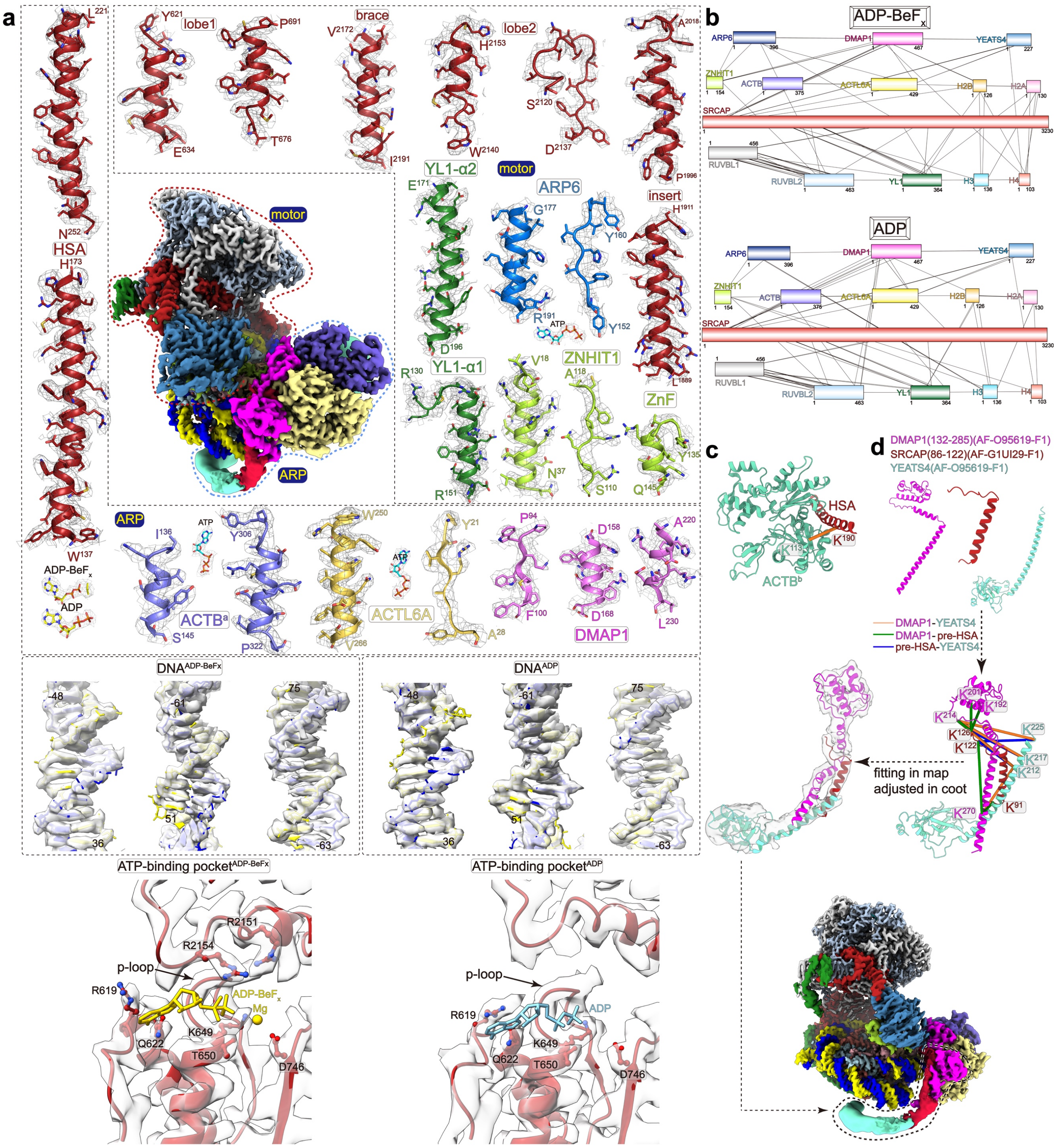

Supplementary Fig. S2: Cryo-EM map and structural models.

a Composite cryo-EM map of the nucleosome-bound SRCAP-C in the ADP-BeF$_x$-bound state is shown in the center. Locally refined cryo-EM maps of representative regions are shown in boxed panels. The structural models fit the maps well, indicating the correct placement of the structural models. **b** Intermodular crosslinks among the nucleosome-bound SRCAP-C in the presence of ADP (bottom) and ADP-BeF$_x$ (top), respectively. The results were obtained from crosslinking mass spectrometry (XLMS). Note that both samples consist of the complex in the apo conformation. **c** Close-up view and representative crosslink (indicated with yellow line) of the HSA-associated ACTB^b. The

model was obtained by docking structural models into locally refined cryo-EM map in the ADP-bound state. **d** The indicated structural models were generated by AlphaFold2 [1] and placed into the cryo-EM map. Representative crosslinks between DMAP1, pre-HSA, and YEATS4 are indicated with lines. The placement of a putative helix bundle was supported by cryo-EM map and XLMS analysis.

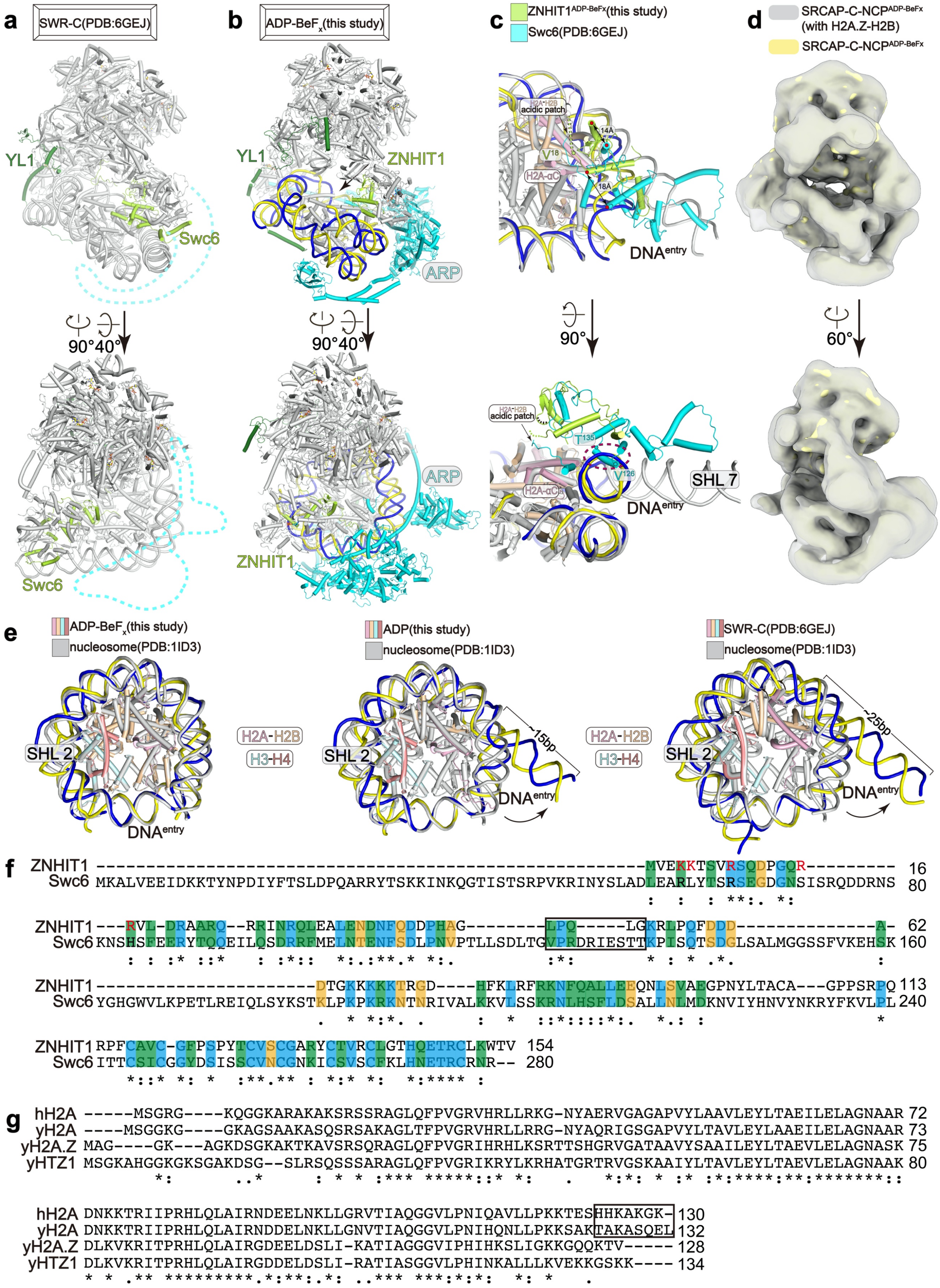

Supplementary Fig. S3: Structure comparison of SRCAP-C and SWR-C.

a-b Structural models of nucleosome-bound yeast SWR-C in the ADP-BeF_x-bound state [2] (a) and nucleosome-bound SRCAP-C in the ADP-BeF_x-bound state (b, this study). Two different views are shown for comparison. **c** Close-up views of the differences between ZNHIT1 and its yeast equivalent subunit Swc6. Nucleosomes are superimposed for comparison. The helix of Swc6 (residues 126-135) clashes with unwrapped DNA, which is absent in ZNHIT1. **d** Superimposition of cryo-EM maps of SRCAP-C-NCP in the ADP-BeF_x-bound state assembled in the presence (grey) and absence (yellow) of H2A.Z-H2B. **e** Superimposition of canonical yeast nucleosome[3] and SRCAP-C-bound nucleosome in the ADP-BeF_x-bound state (left, this study), SRCAP-C-bound nucleosome in the ADP-bound state (middle, this study), and yeast SWR-C-bound nucleosome in the ADP-BeF_x-bound state (right) [2] with differences highlighted. **f** Sequence alignment of ZNHIT1/Swc6. The identical, highly conserved, and less conserved residues are colored in blue, green, and orange, respectively. Key residues in N-terminal region of ZNHIT1 that interacts with acidic patch are colored in red. The box indicates the sequence of the helix of Swc6 which would clash with unwrapped DNA but is absent in ZNHIT1. **g** Sequence alignment of H2A and H2A.Z.

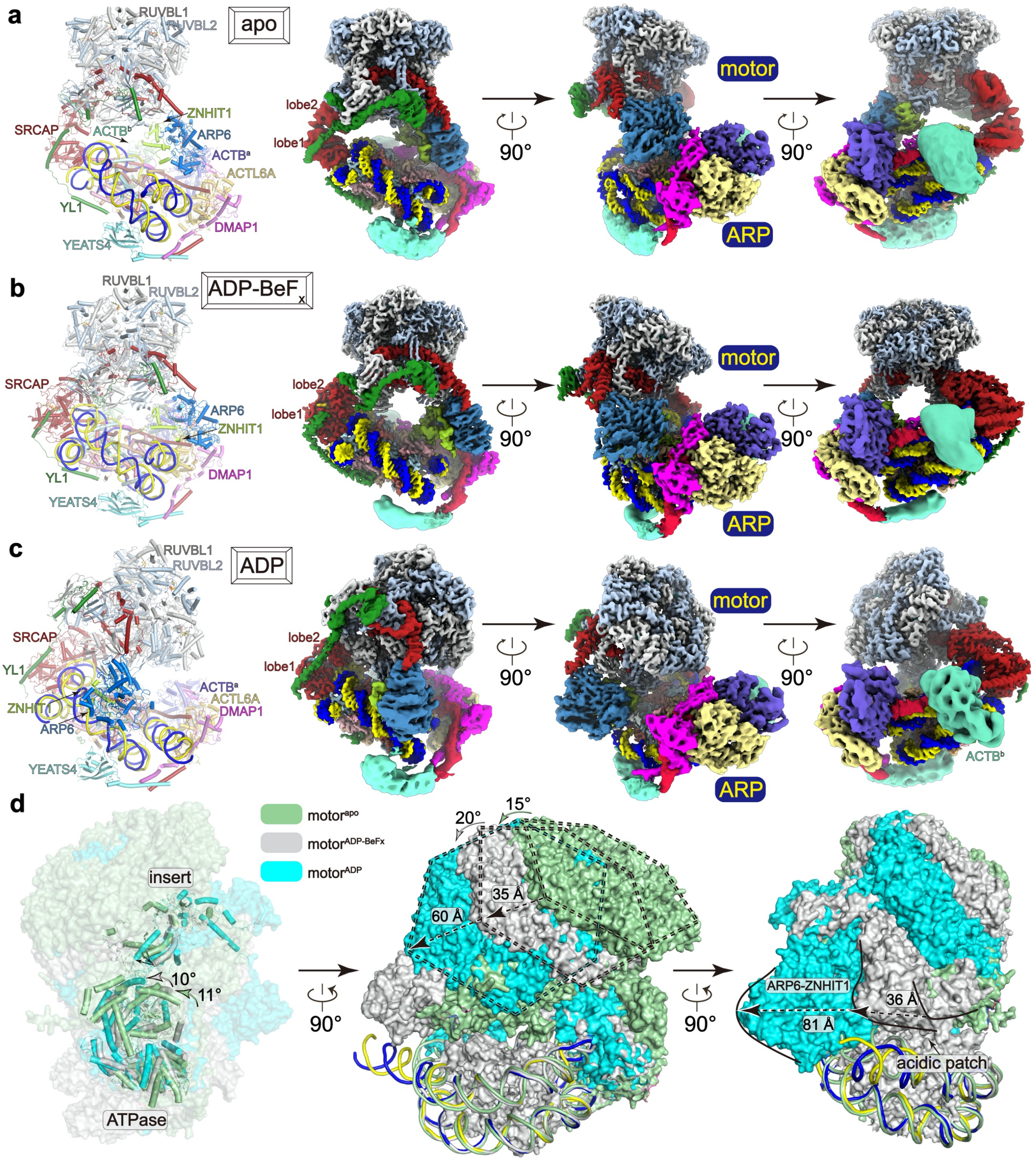

Supplementary Fig. S4: Comparison of overall structures in different nucleotide-binding states. **a-c** Cryo-EM maps in different views and structural models of SRCAP-C-NCP in the apo (a), ADP-BeF$_x$-bound (b), and ADP-bound (c) states. **d** Comparison of the three structures with the nucleosomes superimposed. Conformational differences are highlighted.

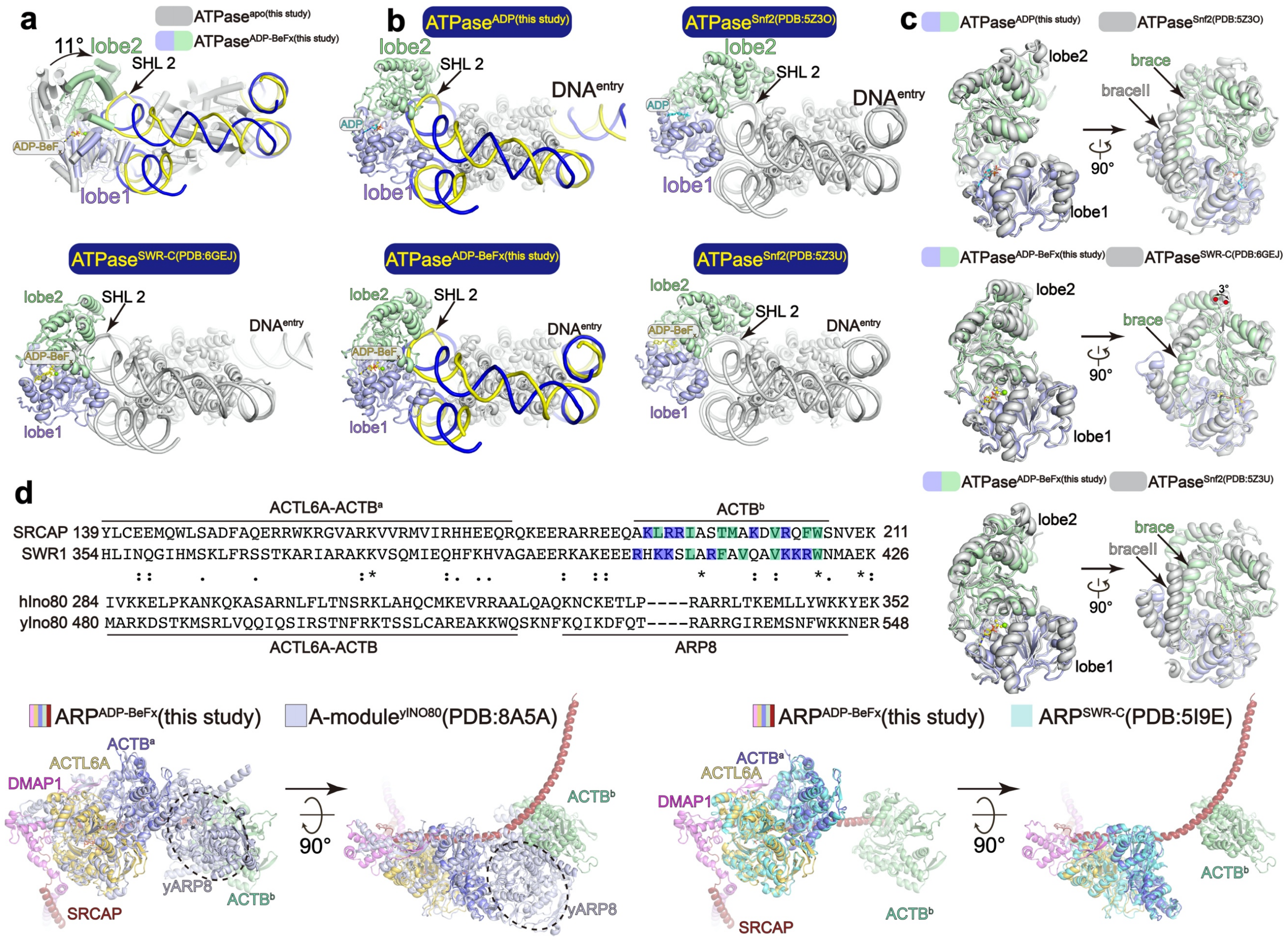

Supplementary Fig. S5: Structure comparison of the ATPases and ARP modules in SRCAP-C and other remodelers.

a Superimposed structures of the nucleosome-bound ATPase of SRCAP-C in the apo state and ADP-BeF$_x$-bound state. The two nucleosomes were superimposed and the nucleosome of the apo form was omitted for clarity. **b** Structural models of nucleosome-bound ATPase in the indicated structures for comparison [2,4]. **c** Comparison of the ATPase domains of indicated structures with the lobe1 superimposed. **d** Superimposition of the ARP modules in the indicated structures for comparison [5,6]. Sequence alignment of SRCAP and Ino80 subunits in humans and yeast shows that the ARP-associated HSA is generally similar in positioning ARP subunits. The ACTB^b-binding motif of HSA is conserved in SRCAP/Swr1 and seems to be equivalent to the ARP8-binding motif in INO80-C.Positively charged residues (Arg and Lys) are colored in blue and hydrophobic residues (Ile, Leu, Trp, Val, Phe, Tyr, and Met) in green in the ACTB^b-binding motif of HSA of SRCAP and SWR1.

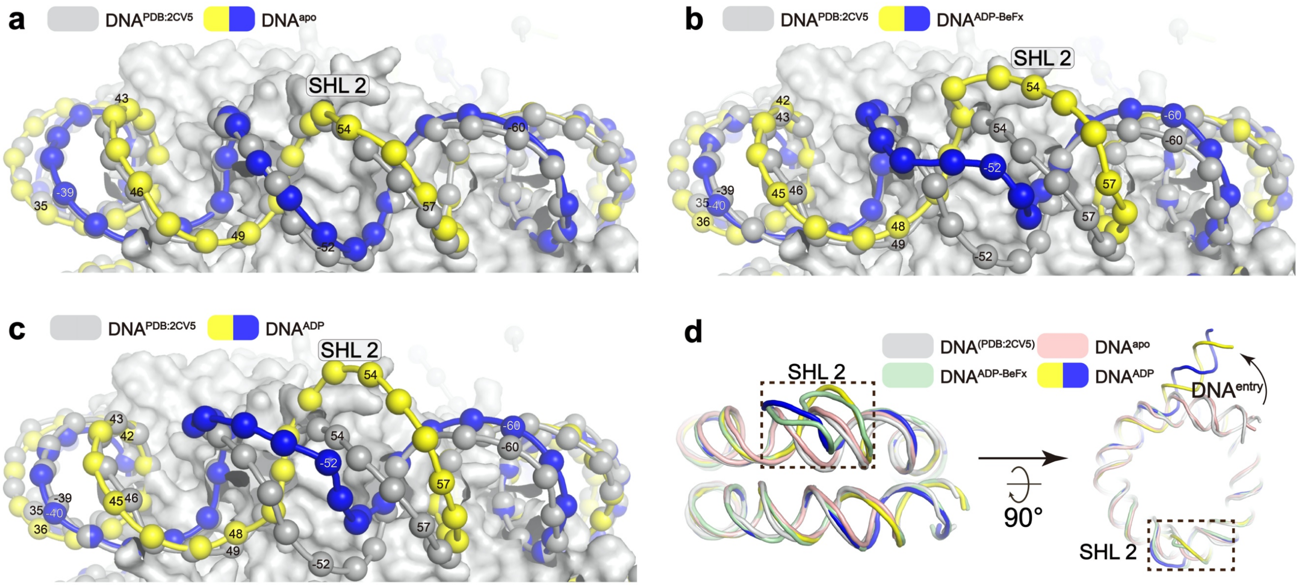

Supplementary Fig. S6: Comparison of nucleosomal DNA in different nucleotide-binding states. **a-c** Comparison of nucleosomes in the SRCAP-C complex structures. The structure of the canonical nucleosome [7] is shown for comparison. **d** Superimposition of the nucleosomal DNA in the apo, ADP-bound, and ADP-BeF_x-bound states. Regions with conformational differences are highlighted.

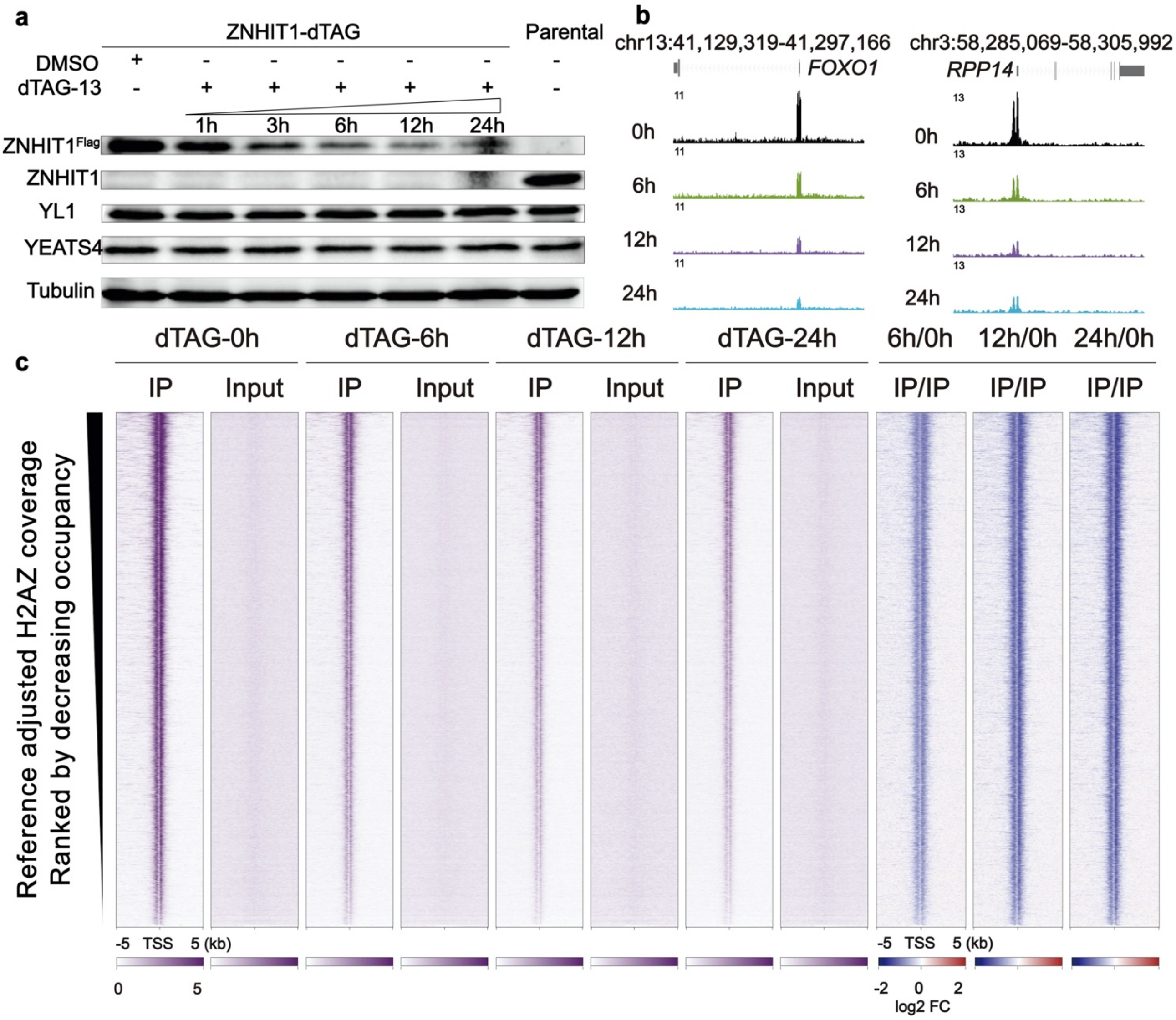

Supplementary Fig. S7: ZNHIT1 is constantly required for histone exchange.

a Detection of ZNHIT1 degradation across varying time intervals. ZNHIT1 represents the endogenous protein in parental DLD1 cells and ZNHIT1^{Flag} represents the protein fused with Flag-FKBP12^{F36V} in ZNHIT1-dTAG cells. Complex composition was validated by western blots of representative SRCAP-C subunits YL1 and YEATS4 in ZNHIT1-dTAG cells. No degradation of the two subunits was observed. **b** Representative track examples showing H2A.Z occupancy in DMSO/dTAG-treated ZNHIT1-dTAG cells. **c** Heatmaps of H2AZ occupancy centered at TSSs of promoters ranked by decreasing occupancy in DMSO/dTAG-treated ZNHIT1-dTAG cells across varying time intervals.

Supplementary Table S1. Data processing and structure refinement.

	SRCAP-C-NCP$^{ADP-BeFx}$				SRCAP-C-NCP^{ADP}				SRCAP-C-NCP^{apo}	
	**Overall** (EMDB-37988) (PDB-8X19)	**ATPase**	**NCP**	**ARP**	**Overall** (EMDB-37990) (PDB-8X1C)	**ATPase**	**NCP**	**ACTB[b]**	**Overall** (EMDB-37984) (PDB-8X15)	**ATPase**
**Data collection and processing**										
Magnification	64,000 x	64,000 x	64,000 x	64,000 x	64,000 x	64,000 x	64,000 x	64,000 x	64,000 x	64,000 x
Voltage (kV)	300	300	300	300	300	300	300	300	300	300
Electron exposure (e^-/Å^2)	50	50	50	50	50	50	50	50	50	50
Defocus range (μm)	-1.0 ~ -2.5	-1.0 ~ -2.5	-1.0 ~ -2.5	-1.0 ~ -2.5	-1.0 ~ -2.5	-1.0 ~ -2.5	-1.0 ~ -2.5	-1.0 ~ -2.5	-1.0 ~ -2.5	-1.0 ~ -2.5
Pixel size (Å)	1.334	1.334	1.334	1.334	1.334	1.334	1.334	1.334	1.334	1.334
Symmetry imposed	C1	C1	C1	C1	C1	C1	C1	C1	C1	C1
Initial particle images (no.)	6,105,534	6,105,534	6,105,534	6,105,534	3,463,395	3,463,395	3,463,395	3,463,395	6,105,534	6,105,534
Final particle images (no.)	475,617	475,617	475,617	192,819	399,935	399,935	399,935	54,761	1,092,654	1,092,654
Map resolution	3.3	3.1	3.0	3.3	3.3	3.1	2.9	4.2	3.3	3.4
FSC threshold	0.143	0.143	0.143	0.143	0.143	0.143	0.143	0.143	0.143	0.143
Map resolution range (Å)	3.0-9.0	3.0-8.0	3.0-8.0	3.0-4.5	3.0-9.0	3.0-8.0	3.0-8.0	3.0-7.5	3.0-9.0	3.0-7.0
**Refinement**										
Model resolution (Å)	3.2				3.2				5.36	
FSC threshold	0.143				0.143				0.143	
Model composition										
Non-hydrogen atoms	97,456				107,579				106,615	
Protein residues	6,372				6,347				6,280	
Nucleotide residues	294				294				257	
Ligands	Mg: 1<br>BeF_3: 1<br>ADP: 7<br>ATP: 2				ADP: 7<br>ATP: 3				ADP: 6<br>ATP: 2	
B factors (Å^2)										
Protein	1.00/314.05/94.71				1.00/314.05/94.51				1.00/314.05/95.40	
Nucleotide	20.00/132.19/43.35				20.00/640.96/98.51				20.00/999.99/857.42	
Ligand	10.44/99.93/57.25				10.49/99.93/58.40				8.73/58.98/31.92	
R.m.s deviations										
Bond lengths (Å)	0.005				0.006				0.009	
Bond angles (°)	0.832				1.189				1.046	
Validation										
MolProbity score	2.94				2.39				2.45	

Clash score	20.92	11.18	13.31
Poor rotamers (%)	7.63	4.72	4.59
Ramachandran plot			
Favored (%)	92.94	95.47	95.50
Allowed (%)	6.6	4.34	4.37
Disallowed (%)	0.48	0.19	0.13

73

Supplementary Table S2. The crosslinking mass spectrometry analysis.

Supplementary Video S1. Composite cryo-EM map and structural model of the nucleosome-bound SRCAP-C in the ADP-BeF_x-bound state.

Supplementary Video S2. Composite cryo-EM map and structural model of the nucleosome-bound SRCAP-C in the apo (no nucleotide) form.

Supplementary Video S3. Composite cryo-EM map and structural model of the nucleosome-bound SRCAP-C in the ADP-bound state.

Supplementary Video S4. Conformational changes of the complex from the ADP-BeF_x-bound state to the ADP-bound state.

Supplementary Video S5. Conformational changes of the complex from the apo form to the ADP-BeF_x-bound state.

Supplementary References:

1 Jumper, J. *et al.* Highly accurate protein structure prediction with AlphaFold. *Nature* **596**, 583-589, doi:10.1038/s41586-021-03819-2 (2021).

2 Willhoft, O. *et al.* Structure and dynamics of the yeast SWR1-nucleosome complex. *Science* **362**, doi:10.1126/science.aat7716 (2018).

3 White, C. L., Suto, R. K. & Luger, K. Structure of the yeast nucleosome core particle reveals fundamental changes in internucleosome interactions. *Embo j* **20**, 5207-5218, doi:10.1093/emboj/20.18.5207 (2001).

4 Li, M. *et al.* Mechanism of DNA translocation underlying chromatin remodelling by Snf2. *Nature* **567**, 409-413, doi:10.1038/s41586-019-1029-2 (2019).

5 Kunert, F. *et al.* Structural mechanism of extranucleosomal DNA readout by the INO80 complex. *Sci Adv* **8**, eadd3189, doi:10.1126/sciadv.add3189 (2022).

6 Cao, T. *et al.* Crystal structure of a nuclear actin ternary complex. *Proc Natl Acad Sci U S A* **113**, 8985-8990, doi:10.1073/pnas.1602818113 (2016).

7 Tsunaka, Y., Kajimura, N., Tate, S. & Morikawa, K. Alteration of the nucleosomal DNA path in the crystal structure of a human nucleosome core particle. *Nucleic Acids Res* **33**, 3424-3434, doi:10.1093/nar/gki663 (2005).
